# A visualizable and widely applicable steric repulsion descriptor for guiding experimental chemistry

**DOI:** 10.1039/d5sc07952g

**Published:** 2026-01-09

**Authors:** Guillaume Hénon Just, Corentin Lefebvre, Akilan Rajamani, Hassan Khartabil, Julien Pilmé, Éric Hénon

**Affiliations:** a Ecole Nationale des Ponts et Chaussées, Institut Polytechnique de Paris, Département IMI 6 et 8 Avenue Blaise-Pascal, Cité Descartes, Champs-sur-Marne 77455 Marne-la-Vallée France; b Laboratoire de Glycochimie et des Agroressources d’Amiens, UR 7378, Université de Picardie Jules Verne 10 rue Baudelocque Amiens France; c Institut de Chimie Moléculaire de Reims (ICMR) UMR CNRS 7372, Université de Reims Champagne-Ardenne Moulin de la Housse BP 1039 51687 Reims CEDEX 2 France eric.henon@univ-reims.fr; d Laboratoire de Chimie Théorique, UMR 7616, Sorbonne Université Paris France

## Abstract

While steric effects fundamentally shape molecular behavior and are experiencing renewed interest across chemistry, visualizing where they occur remains challenging. Indeed, steric interactions have traditionally been depicted in a qualitative, often sketchy manner. We present SELF (Steric Exclusion Localization Function), which enables the quantification, atomic resolution, and visualization of steric repulsion in three dimensions (whether between molecules or between fragments of a single molecule) directly from quantum-mechanical calculations. Accessible through the user-friendly IGMPlot software, SELF transforms abstract quantum-mechanical concepts into visual insights that chemists can immediately interpret and apply. We illustrate SELF's versatility across diverse chemical systems: from understanding atropisomerism in pharmaceutical scaffolds to rationalizing selectivity in catalysis through steric effects of phosphorus ligands in organometallic chemistry. Unlike traditional quantum-mechanical methods which condense the characteristics of steric effects into a single number, SELF provides unprecedented spatial resolution of steric clashes while maintaining rigorous quantitative analysis. This approach bridges the gap between theoretical concepts and practical chemical understanding, making sophisticated quantum-mechanical analysis accessible to the broader chemical community. We hope this tool will prove valuable for chemical design, research applications, and teaching steric effects.

## Abstract 2

SELF makes steric effects visible and measurable. Built from quantum calculations, it maps steric repulsion in 3D, from drug scaffolds to catalysis, turning abstract theory into intuitive, practical chemical insight.

## Graphical abstract

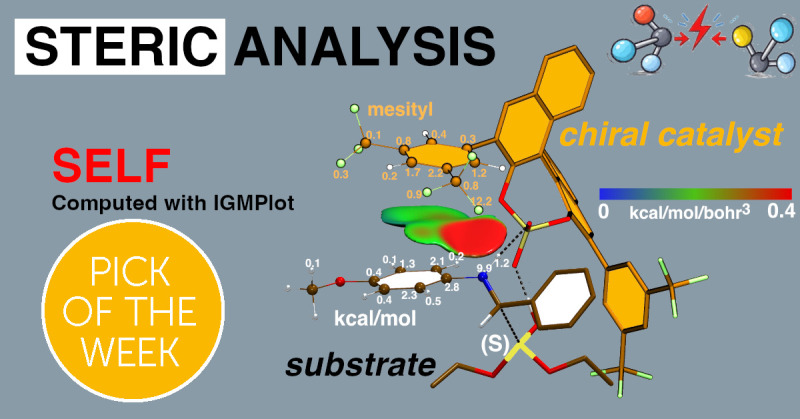

## 1 Introduction

Few concepts in chemistry are as universally invoked yet as poorly visualized as steric effects. Here, we introduce SELF (Steric Exclusion Localization Function), which brings quantification, atomic resolution, and three-dimensional visualization to this fundamental phenomenon.

The pioneering work of Noyori on asymmetric hydrogenation demonstrates^1,2^ how subtle steric interactions can contribute to reaction outcomes. More broadly, such steric effects play a key role throughout chemistry.^3–8^ While the IUPAC definition of steric effects is useful for dissecting them into contributions: non-bonded repulsions, bond-angle strain, and bond stretches or compressions, it offers no insight into their fundamental physical origin. However, it is now widely accepted that, at the atomic scale, this origin lies primarily in Pauli repulsion, particularly at short range.^9^

The Pauli exclusion principle, prohibiting electrons with the same spin from occupying the same spatial region, underlies the steric effect, which is pivotal in shaping modern chemical understanding and chemical intuition. When molecules approach each other, this quantum-mechanical (QM) constraint manifests itself as a repulsive force. Despite their ubiquity, steric effects are most generally invoked qualitatively in chemical discourse and literature without explicit spatial or quantitative characterization.

Recent investigations have revealed the Pauli repulsion's significance to be far greater than previously assumed. Among the wealth of references exploring steric effects, Bickelhaupt and collaborators demonstrated its dominant role in Lewis acid-catalyzed Diels–Alder reactions, and proposed the general concept of Pauli repulsion-lowering in catalysis.^10^ Similarly, recent studies on σ-hole interactions^11^ and π-stacking in aromatic systems^12^ have challenged conventional interpretations by highlighting the critical role of exchange repulsion.

Regarding steric effect modeling, empirical schemes such as Taft's^3^ have limited predictive value for unprecedented substituents, serving primarily as descriptive correlations rather than true predictive tools. Geometry-based descriptors such as topographic steric maps and buried volume^13^ rely explicitly on van der Waals radii, while Sterimol^14^ parameters provide a simple geometric description of steric effects. Beyond classical force fields, including polarizable models such as the Drude oscillator,^15^ current quantum-mechanical-based methods, while powerful, present significant limitations in estimating Pauli repulsion. Among them, Energy Decomposition Analysis (EDA)^16–18^ and Symmetry-Adapted Perturbation Theory (SAPT)^19^ provide a quantification of Pauli repulsion but lack spatial information about where these interactions occur: they condense the characteristics of steric effects into a single number. Local partitioning schemes such as fp-LED^20^ or atomic SAPT^21^ (limited to intermolecular contexts) provide fragment- or atom-resolved Pauli repulsion contributions, yet none of these approaches offers true spatial resolution of steric interactions in three-dimensional space. Natural bond orbital (NBO) analysis^22^ offers another perspective by decomposing exchange repulsion into contributions from localized functional groups, but this resolves interactions in chemical space rather than in real space. Yet, Pauli repulsion is intrinsically a local effect, making this lack of spatial resolution a fundamental limitation for understanding molecular interactions. In other respects, the Interacting Quantum Atoms (IQA) density-based approach^23–25^ extracts steric effects through an indirect route, by quantifying intra-atomic deformations rather than direct inter-atomic repulsions, demonstrating that even the fundamental strategy for extracting such effects from electron density (ED) remains an open question. These approaches typically require multiple calculations (separate monomers, complex) and demand sophisticated theoretical expertise, limiting their accessibility. Moreover, analyzing intra-substituent steric effects within a molecule using fragmentation requires breaking covalent bonds, often leading to open-shell species that complicate traditional QM calculations. In other respects, steric maps obtained by using a chemical probe (like fluoride) from different directions introduce arbitrary bias, and the associated exhaustive grid scanning makes this approach computationally demanding.^9^ Distinct from the above approaches, the electron density-based NCI index^26^ characterizes the presence of bonding or non-bonding non-covalent interactions; however, the systematic association of positive values of *λ*_2_ (second eigenvalue of the ED hessien) with steric repulsion appears to be an overstatement that has been recently critically examined in the literature.^27^

We introduce here the Steric Exclusion Localization Function (SELF), a method that characterizes and spatially resolves regions of steric repulsion between molecules or molecular fragments of a molecule. SELF builds on the core concept underlying the Electron Localization Function (ELF),^28,29^ namely, the Pauli Kinetic Energy Excess (KEE): the kinetic energy increase that occurs when same-spin electrons occupy the same region. Although Pauli energy has previously been employed to characterize steric energy,^30–32^ these attempts treated it as a global molecular property, which inherently limits their ability to isolate specific steric repulsion between fragments or molecules. To the best of our knowledge, the approach presented in this work represents the first systematic attempt to decompose Pauli kinetic energy excess into distinct intra-fragment and inter-fragment contributions. The key innovation of SELF lies in decomposing the Pauli KEE density *C*(**r**) into these distinct contributions, with the inter-fragment term *C*(**r**)_**22**_ directly mapping steric interactions.

SELF offers several advantages: (1) it requires only a single electronic structure calculation on the complete system as input, (2) it provides both visual representation and quantitative analysis of Pauli repulsion, (3) it enables atomic-level decomposition of steric effects, (4) it enables steric repulsion between molecular fragments within and between molecules to be readily described within a unified framework. The method is implemented in the freely available IGMPlot software,^33^ making sophisticated steric analysis accessible to the broader chemical community.

After introducing the theoretical framework through the intuitive water dimer example, we establish SELF's physical foundations using noble gas dimers. We then demonstrate its ability to capture the anisotropic nature of steric repulsion in molecular complexes, before showcasing practical applications: identifying the steric origins of stereoselectivity in organocatalysis and unveiling the hidden dance of atropisomerism in pharmaceutical scaffolds. Finally, we broaden our scope to coordination chemistry with an analysis of Ni(CO)_3_L phosphine complexes, revisiting Tolman's classical framework on steric effects of phosphorus ligands.

## 2 Results and discussion

### 2.1 Methodology

The SELF methodology quantifies steric repulsion by isolating the Kinetic Energy Excess (KEE) that arises when like-spin electrons from different molecular fragments are forced into spatial proximity. The theoretical foundation rests on the Pauli KEE function *C*(**r**), already mentioned in Levy and Ou-Yang's work^34^ and employed in the Electron Localization Function (ELF) theory:^28,29^

1 *C*(**r**) = *G*(**r**) − *G*_W_(**r**)

where *G*(**r**) is the positive kinetic energy density:2
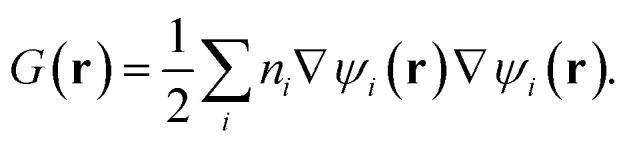
Here *ψ*_*i*_(**r**) denotes either molecular orbitals (HF/DFT) or natural orbitals with fractional occupancies *n*_*i*_ (correlated methods), following the generalization previously established for ELF theory.^35,36^*G*_W_(**r**) is the von Weizsäcker kinetic energy density^37^ for a bosonic system (not experiencing Pauli repulsion):3
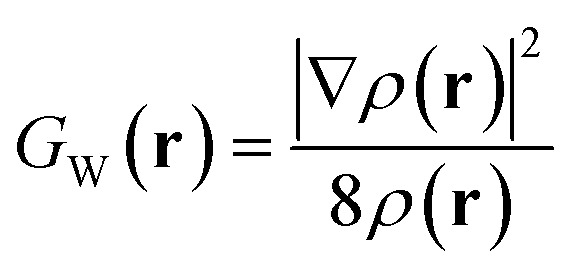
where *ρ*(**r**) is the electron density (ED). This quantity *C*(**r**) measures the increase in local kinetic energy due to the Pauli exclusion principle.

**Fig. 1:**
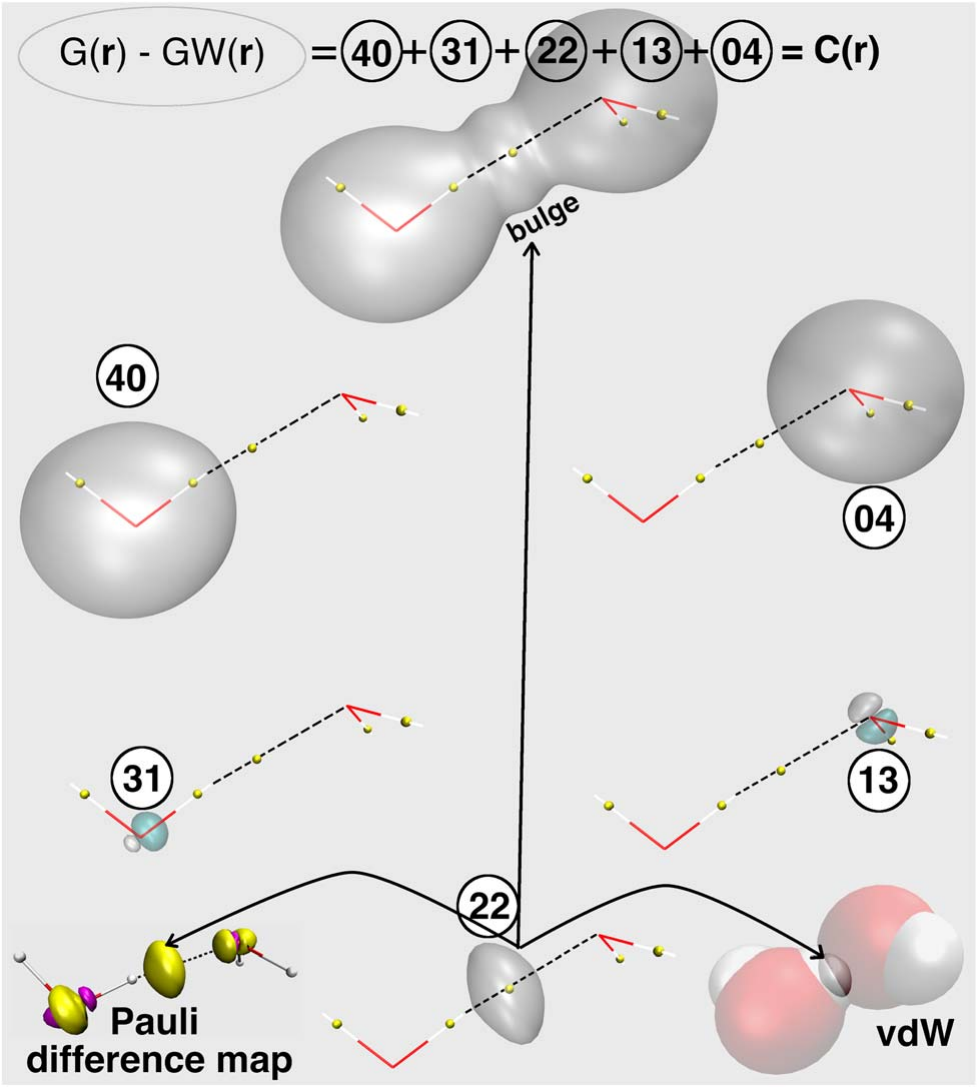
Spatial decomposition of the total Pauli kinetic energy excess (KEE) in a compressed water dimer using the SELF approach. The KEE, *C*(**r**) = *G*(**r**) − *G*_W_(**r**), is partitioned into five components *C*(**r**)_**XY**_ according to the distribution of the four atomic orbital indices abcd (in the summation of eqn (5)) over the two molecules. *C*(**r**)_**40**_ and *C*(**r**)_**04**_ arise from single-molecule terms, while *C*(**r**)_**31**_, *C*(**r**)_**13**_ and *C*(**r**)_**22**_ capture inter-molecule interactions, with *C*(**r**)_**22**_ representing the leading steric repulsion term between fragments. Translucent iso-surfaces of these terms (*C*(**r**) and its five components) are shown at +0.01 a.u. (gray) and −0.01 a.u. (cyan). Calculations were performed at the (DFT) M06-2X/def2-TZVP level of theory on the dimer geometry taken from the S22 data set,^38^ compressed in H-bond axis to 90%. Yellow spheres indicate bond critical points (*i.e.*, characteristic points from Bader's Atoms in Molecules (AIM) theory indicating the presence of a local bonding situation, shown here to locate the hydrogen bond relative to the SELF isosurfaces). Bottom right: van der Waals (vdW) representation of the water dimer highlighting how the *C*(**r**)_**22**_ iso-surface emerges at the intersection of hydrogen and oxygen atomic volumes. Bottom left: *C*(**r**) difference map between dimer and isolated monomers (extracted from Fig. S1 in SI), revealing KEE redistribution upon complexation: yellow positive (purple negative) values indicate local KEE accumulation (depletion).

Considering a molecular system composed of two fragments, the global function *C*(**r**) encompasses both the Pauli repulsion of electrons within each fragment (naturally present in isolated fragments) and the repulsion effects that emerge when fragments approach each other, the latter representing only a subset of the total KEE. A manifestation of this subset is clearly visible, even without a formal decomposition scheme for *C*(**r**), as a characteristic bulge in the *C*(**r**) envelope within the interfacial region for the compressed water dimer (see Fig. 1), which appears to be associated with electron cloud overlap and the resulting volume exclusion arising from short-range steric repulsion.

To develop our decomposition scheme, we examined how Pauli kinetic energy *C*(**r**) evolves spatially upon water dimer formation. A ‘Pauli difference map’, constructed by subtracting superposed *C*(**r**) values of isolated monomers from the dimer's *C*(**r**) (Fig. S1 in SI), reveals two contributions: an inter-fragment component between molecules (matching the bulge in Fig. 1), directly linked to steric repulsion, and intra-fragment adjustments within each molecule. The inter-fragment contribution captures the volume exclusion routinely identified in experimental literature as the hallmark of steric repulsion. However, this numerical approach requires cumbersome cube file manipulations and cannot address steric effects between groups within the same molecule. This prompted us to develop an analytical approach for isolating the intermolecular contribution present in *C*(**r**).

When molecular orbitals *ψ*_*i*_ are expressed as linear combinations of atomic orbitals (AO) *ϕ*_a_, the Pauli KEE can be rigorously partitioned. The first step involves bringing terms of *C*(**r**) to a common denominator, yielding the unified expression:4
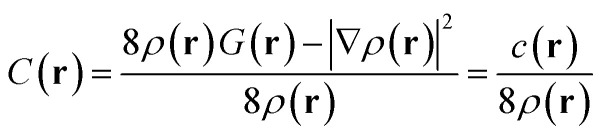


for *ρ*(**r**) ≠ 0.

Importantly, precisely like *C*(**r**), *c*(**r**) approaches zero in the absence of Pauli interactions; otherwise it has positive values. It is a subtle point but conceptually very important. Indeed, since *ρ*(**r**) is strictly positive, and *C*(**r**) is non-negative, *c*(**r**) must also be non-negative. Then, in interfragment regions where *ρ*(**r**) remains moderate (away from nuclear positions), *C*(**r**) → 0 implies *c*(**r**) → 0, making *c*(**r**) an equally effective probe of Pauli repulsion. Ultimately, *c*(**r**) can be interpreted as the Fermi hole curvature,^39,40^ the cornerstone of Kohout's development of the Electron Localizability Indicator (ELI)^41^ for electron localization analysis.

After algebraic manipulation (detailed derivation in SI Section S5), we obtain a four-index summation over atomic orbitals:5
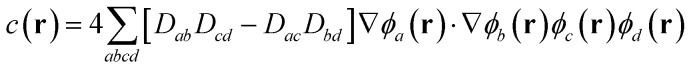
where *D*_*ab*_ are elements of the density matrix:6
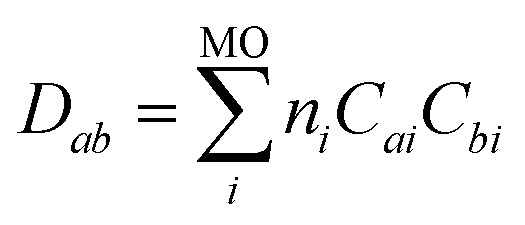


Noticeably, *D*_*ab*_*D*_*cd*_ and *D*_*ac*_*D*_*bd*_ only differ by the exchange of two AOs. When two molecules X and Y (or fragments of a single molecule) interact, we can split *c*(**r**) in eqn (5) (see also Fig. 1) according to the number of AO participating to each fragment:

7 *c*(**r**) = *c*(**r**)_**40**_ + *c*(**r**)_**04**_ + *c*(**r**)_**31**_ + *c*(**r**)_**13**_ + *c*(**r**)_**22**_

and hence:

8 *C*(**r**) = *C*(**r**)_**40**_ + *C*(**r**)_**04**_ + *C*(**r**)_**31**_ + *C*(**r**)_**13**_ + *C*(**r**)_**22**_

The term *C*(**r**)_**22**_ represents elements of *c*(**r**) where two atomic orbitals (out of the four) belong to the first molecule X, and the remaining two atomic orbitals belong to the second molecule Y. Contributions *C*(**r**)_**31**_ and *C*(**r**)_**13**_ arise when three orbitals (out of the four) belong to one fragment and the remaining orbital belongs to the other fragment. In contrast, the terms *C*(**r**)_**40**_ and *C*(**r**)_**04**_ represent purely intra-fragment contributions. This decomposition merely requires the definition of two molecular fragments. Fig. 1 illustrates the decomposition of the excess of kinetic energy *C*(**r**) in the compressed water dimer into its five distinct contributions.

As expected, the intramolecular components (**40** and **04**) in Fig. 1 are localized within each water molecule and dominate the total integrated KEE (Table 1), reflecting strong intra-molecular Pauli repulsion. Table 1 also presents the sensitivity on grid-size of the integrated contributions to the Pauli KEE. Components **40** and **04** exhibit significant sensitivity to the chosen grid spacing, necessitating a fine grid resolution for convergence. This sensitivity results from contributions localized in the core regions near the nuclei, where the kinetic energy density is highest. Remarkably, each of these two dimer's components **40** and **04** display a near-perfect match with the corresponding *C*(**r**) features of the isolated monomers (see Fig. S2 in ESI). Components **31** and **13**, though intermolecular in origin, spatially appear in Fig. 1 within each fragment with small negative and positive values (reflecting local intra-fragment KEE adjustment upon interaction, the overall Pauli KEE *C*(**r**) remaining strictly positive throughout space). Importantly, **31** and **13** contributions concentrate around atomic centers within each fragment, not in the inter-fragment region. Moreover, the **31** and **13** components remain around one to two orders of magnitude smaller than the dominant inter-fragment term **22** throughout the thermally accessible configuration space (see Tables S1–S5 in SI).

**Table 1:** Decomposition of the kinetic energy excess *C* arising from Pauli repulsion in the compressed water dimer. The spatial components *C*(**r**)_**XY**_ shown in Fig. 1 are here integrated over the entire numerical grid, yielding total contributions *C*_**XY**_ (in Hartree) to the overall KEE. The influence of the grid stepsize on these integrated values is reported. Calculations were performed at the (DFT) M06-2X/def2-TZVP level of theory on the dimer geometry taken from the S22 data set,^38^ compressed in H-bond axis to 90%

Grid stepsize (Å)	**40**	**04**	**31**	**13**	**22**	*C*
0.200	18.39493	18.98137	−0.01391	−0.01424	0.17869	37.52684
0.100	18.59413	18.57978	−0.01361	−0.01451	0.17867	37.14579
0.050	18.61962	18.58616	−0.01359	−0.01449	0.17869	37.35639
0.025	18.62281	18.58775	−0.01359	−0.01450	0.17869	37.36116
0.010	18.62122	18.58775	−0.01359	−0.01450	0.17869	37.35957

Interestingly, component **22** emerges as a natural descriptor for intermolecular steric exclusion effects. It is positive, spatially localized in the overlap region, it precisely matches the bulge in total *C*(**r**) and, crucially, shows remarkable robustness to grid resolution, with negligible dependence on the grid spacing (see Table 1). Two additional observations reinforce the physical foundation of this descriptor. First, the iso-surface of component **22** lies exactly at the intersection of the van der Waals spheres of H and O atoms in the compressed hydrogen-bonded water dimer (see Fig. 1), confirming its association with the volume exclusion arising from Pauli repulsion. Second, component **22** aligns perfectly with the interfragment contribution that appears in between the two molecules in the map derived from the manually computed KEE difference between dimer and monomers (see Fig. 1 and S3 in SI), a parameter-free and purely numerical reference that provides an unbiased benchmark for Pauli repulsion.

Based on the aforementioned results, we opted for component **22** as our framework for capturing the spatial distribution of steric exclusion between two interacting fragments and its quantification.

The so-called Steric Exclusion Localization Function (SELF) stems from this development:9
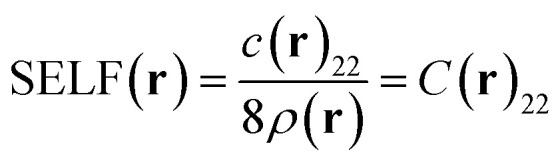


**Fig. 2:**
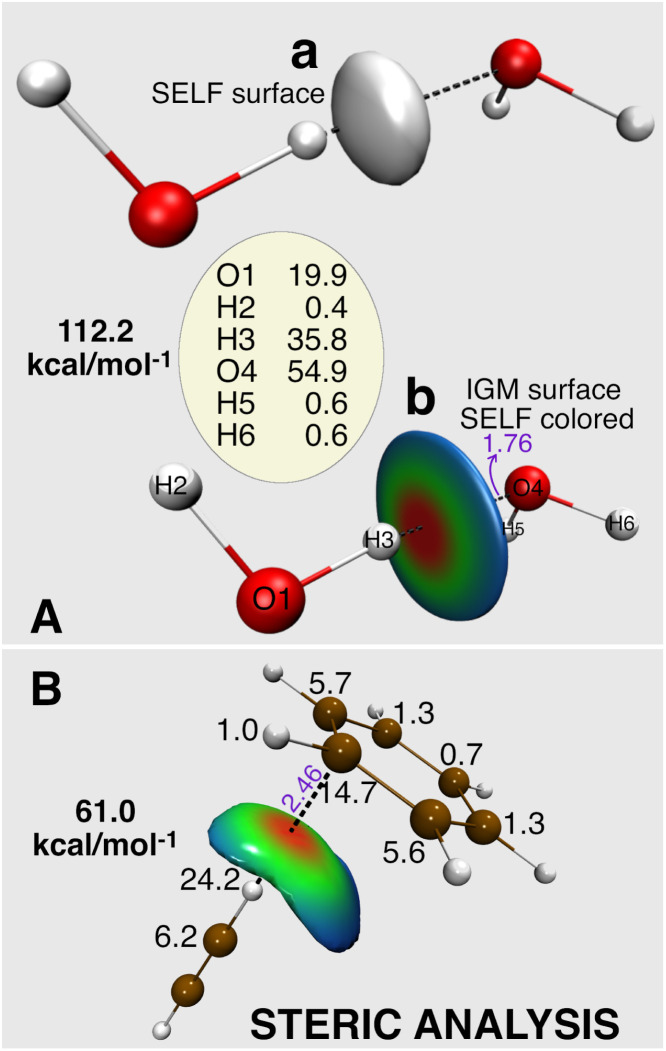
(A) Two graphical representations of the spatial distribution of the steric exclusion between two water molecules in a non-equilibrium geometry, obtained using the SELF(**r**) descriptor: (a) SELF(**r**) = 10 kcal mol^−1^ bohr^−3^ isosurface, (b) 0.8 a.u.^−1^*δg*^inter^/*ρ* isosurface colored by the SELF(**r**) values in the range 0 to 15 kcal mol^−1^ bohr^−3^ on a BGR color scale (red = large steric repulsion). Atomic decomposition in kcal mol^−1^. Geometry taken from the S22 data set,^38^ shifted in H-bond axis to 90% of the original H-bond distance. (B) Steric analysis of C_2_H_2_ in complex with C_6_H_6_ in a compressed, non-equilibrium geometry inducing atomic clashes; 0.59 a.u.^−1^*δg*^inter^/*ρ* isosurface colored by the SELF(**r**) values in the range 0 to 2.8 kcal mol^−1^ bohr^−3^ on a BGR color scale; atomic decomposition in kcal mol^−1^. Level of theory: DFT M06-2X/def2-TZVP. Distances in Å.

A direct SELF(**r**) iso-surface (Fig. 2A,a, water dimer), while intuitive, only captures a single threshold value. To visualize steric exclusion effects spatially (Fig. 2A,b and B), we prefer color-coding the *δg*^inter^/*ρ* iso-surface derived from the Independent Gradient Model (IGM) (see IGM references for more details)^42,43^ with SELF(**r**) values. This approach is particularly effective as the IGM *δg*^inter^/*ρ* descriptor inherently delineates the spatial domains where ED from different fragments overlap, precisely the areas most susceptible to steric repulsion effects. This representation offers a crucial chromatic BGR gradient that vividly maps variations in steric repulsion intensity across the interface between fragments, with red indicating maximum steric repulsion.

To quantify steric effects between fragments, SELF is integrated over the entire grid, yielding the overall steric effect referred to as iSELF10
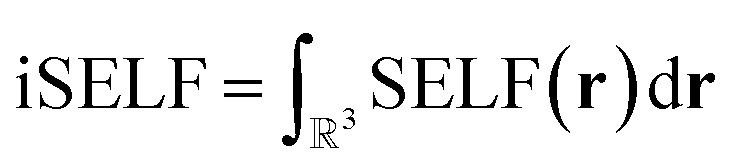


Finally, the orbital-level formulation (eqn. (5) and (9)) enables atomic-level decomposition of steric effects (see Fig. S4 in SI), revealing which atoms drive repulsion, crucial for rational molecular design. Applied to the compressed water dimer (numbers on Fig. 2A), the expected concentration of steric effects on the three hydrogen bond atoms validates our approach. We anticipate this tool will provide more novel insights into the atomic contributions that drive steric hindrance in the context of organic chemistry where it is often invoked, as illustrated below in organocatalysis.

### 2.2 Physical foundations of SELF from SELF-EDA comparison

**Fig. 3:**
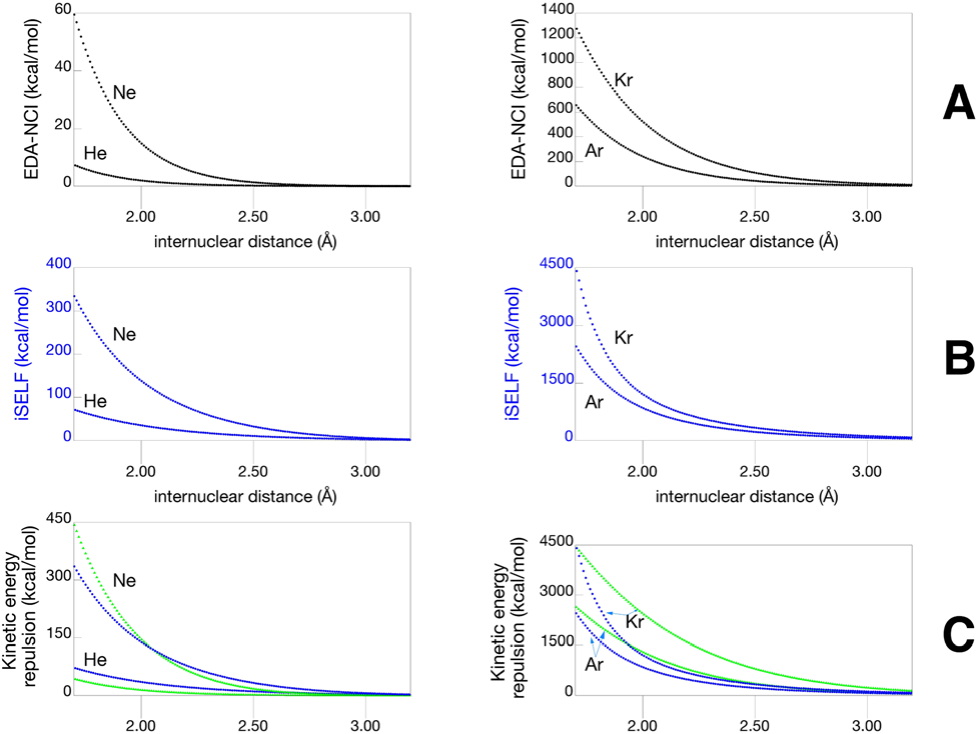
Steric repulsion in He, Ne, Ar and Kr homodimers; (A) EDA-NCI exchange repulsion (B) iSELF; (C) iSELF and kinetic repulsion contribution of EDA-NCI (green). DFT B3LYP/6-31G* Level of theory.

Fig. 3 compares SELF with the gold-standard EDA-NCI methodology^44^ (a traditional energy decomposition analysis) for noble gas dimers (He, Ne, Ar, Kr). Two key conceptual differences emerge. First, while EDA (black color) and iSELF (blue) curves exhibit similar shapes, their energy scales differ significantly (panels A and B). This is because EDA's exchange-repulsion term contains two opposing contributions arising from antisymmetrization of the total electronic wavefunction: a repulsive kinetic component and an attractive exchange component,^45,46^ whereas SELF captures only the repulsive part. Indeed, when we isolate the purely kinetic repulsive term of the Pauli repulsion from EDA (panel C, green), the energy scales become fully compatible, validating the SELF theoretical framework. Nevertheless, a difference persists between EDA and SELF curves. This difference involves electron density treatment: EDA calculates Pauli repulsion between frozen, unrelaxed fragment densities, whereas SELF operates on the fully relaxed complex, inherently capturing all electronic relaxation effects (charge transfer and polarization) while assessing Pauli repulsion. The observed growing discrepancy along the series reflects the dual role of atomic size: larger electron clouds enhance density overlap at short range, while higher polarizability amplifies the electronic relaxation effects captured by SELF but absent from EDA-NCI's frozen-density approximation, both effects culminating in krypton. Nevertheless, both approaches provide consistent insights into Pauli repulsion, confirming SELF as a computationally efficient alternative.

From a methodological standpoint, unlike EDA or SAPT schemes, the SELF method does not necessitate monomer computations, relying solely on a single dimer wave function input file. The simplicity of the SELF workflow remains advantageous to evaluate steric effect between closely packed fragments in a single molecule (see below). The minimalist input format for SELF calculations, illustrated with a working example, is detailed in the SI (Section S8).

### 2.3 Rotational anisotropy of interfragment repulsion

A fundamental criterion for assessing the physical realism of any interaction model is its ability to capture the anisotropy of steric repulsion, a feature that classical force fields inherently lack.^47^

**Fig. 4:**
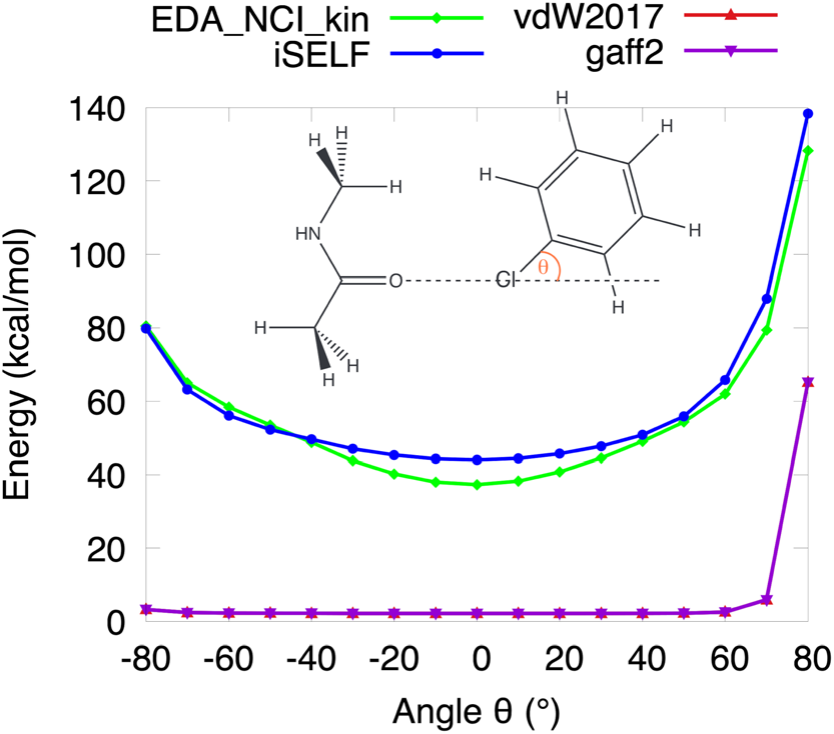
Repulsion energy analysis as a function of the tilt angle for *N*-methylacetamide⋯chloro-benzene dimer, O⋯Cl distance fixed at 3.00 Å. EDA-NCI kinetic component (green), iSELF (blue), vdW2017 (red) and gaff2 (purple), with the two latter curves nearly coincident and then visually hardly distinguishable. DFT B3LYP/6-31G* level of theory.

This anisotropy, arising from the non-spherical distribution of electron density around atoms, manifests as direction-dependent variations in repulsive interactions. To test whether SELF captures this essential feature, we examined the *N*-methylacetamide…chlorobenzene dimer as the O⋯Cl–C angle varies (Fig. 4). SELF successfully captures the anisotropic character of steric repulsion, with notable sensitivity near 0°, exhibiting strong concordance with the EDA-NCI kinetic component, despite originating from fundamentally different conceptual foundations. In stark contrast, classical force fields (vdW2017,^48^ gaff2 ^49^) yield nearly flat curves, lacking this essential physical feature. Although absolute values may differ between SELF and EDA-NCI (particularly in strongly repulsive configurations, see Fig. S5 in SI) their anisotropic profiles show consistent trends. This difference in magnitude is physically grounded: in the high-repulsion regime, electronic relaxation reduces the overlap between fragment electron clouds, which significantly impacts the Pauli repulsion in SELF, whereas the frozen-density approximation in EDA-NCI does not account for this relief. Note that the same reasoning applies to electron-rich systems such as the krypton dimer (Fig. 3), for which the largest SELF/EDA-NCI difference was observed among the rare gas series. Despite this offset, the shared anisotropic signature underscores both the physical basis and practical robustness of the SELF approach.

### 2.4 Organo-catalysis

**Fig. 5:**
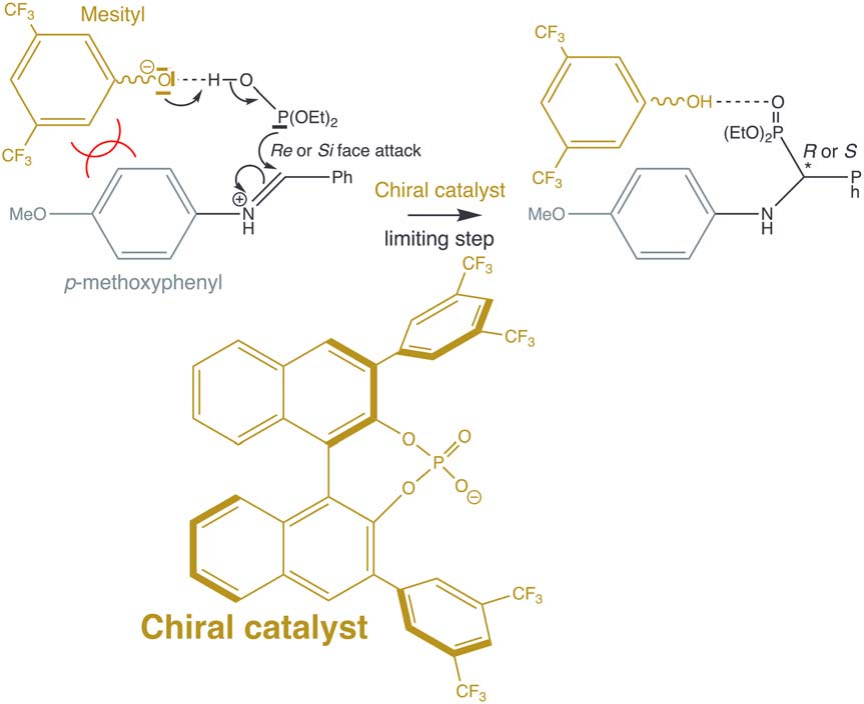
Reaction scheme for the rate limiting step (nucleophilic addition) of the enantioselective hydrophosphonylation. The stereogenic carbon in the final product is denoted by an asterisk. The steric clash is symbolized in the figure by two intersecting arcs between the two concerned fragments. For the sake of clarity, a wavy line is employed, which denotes connection of the mesityl group to the remainder of the catalyst structure.

**Fig. 6:**
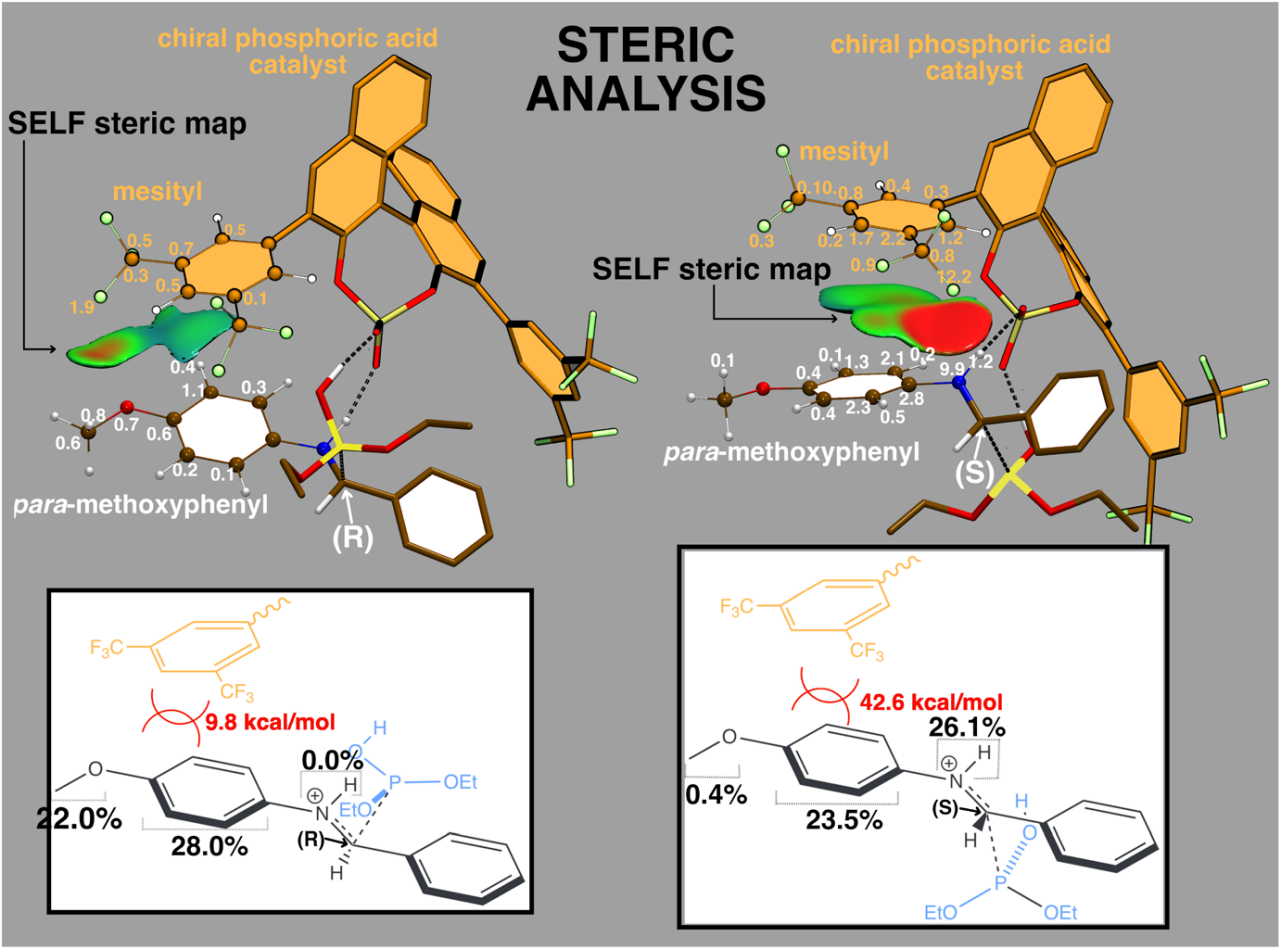
Steric analysis of the TS structures (*R*) and (*S*) for the rate limiting step (nucelophilic addition) of the enantioselective hydrophosphonylation assisted by a chiral catalyst (colored in orange). Fragment 1 (mesityl) = C_6_H_3_(CF_3_)_2_; Fragment 2 = CH_3_O–C_6_H_4_–NH. Fragments used in the SELF analysis are represented in a ball-and-stick model, with atomic contributions to the steric interaction between them indicated next to each atom. For the sake of clarity, in the 2D chemical structure, a wavy line is employed, which denotes connection of the mesityl group to the remainder of the catalyst structure, and the steric clash is symbolized in the figure by two intersecting arcs between the two concerned fragments. The isosurface of the *δg*^inter^/*ρ* IGM descriptor (0.6 a.u.^−1^) highlights electronic clashes between fragments, with colors representing the SELF values to quantify the steric effect. A BGR color scale is applied to represent the SELF descriptor values (blue = no steric interaction, red = large steric interaction). Same *δg*^inter^/*ρ* isovalue and same SELF color range [0 : 0.4 kcal mol^−1^ bohr^−3^] were used for both TS to ensure a consistent comparison of steric interactions. Bottom: contribution of three atomic groups of the substrate (total = 50%) interacting with the mesityl moiety of the catalyst. The integrated iSELF score is reported in orange. Atomic contributions are reported in kcal mol^−1^. Level of theory: DFT B3LYP/6-31G(d).

Steric effects are crucial in organocatalysis,^6^ yet the literature often treats them superficially with terms lacking quantitative analysis. SELF offers a simple and robust framework for atomic-level quantification of these effects, as illustrated by the asymmetric synthesis of α-amino phosphonates *via* enantioselective hydrophosphonylation.^50^ In this system, Shi and Song identified the nucleophilic addition of HOP(OEt)_2_ to an iminium salt as rate-limiting (Fig. 5). SELF analysis reveals the atomic contributions underlying the steric repulsion that governs stereoselectivity in this transformation (Fig. 6).

The authors proposed, without quantum characterization, that (*R*)-selectivity (43% ee) arises from different steric clashes in the two transition states between the catalyst's mesityl group (orange) and the substrate's *p*-methoxyphenyl moiety (Fig. 5). First and foremost, the SELF spatial representation provides a precise localization of steric interactions, often assumed but rarely visualized with such clarity (Fig. 6).

iSELF analysis confirms the authors' hypothesis: the (*S*)-approach experiences substantially stronger steric repulsion (42.6 kcal mol^−1^) than the (*R*)-approach (9.8 kcal mol^−1^).

More surprisingly, SELF reveals interactions the authors missed entirely. In the (*R*)-TS, the *p*-methoxyphenyl group interacts uniformly with the mesityl (methoxy: 22%, phenyl: 28%). However, in the (*S*)-TS, while the methoxy barely interacts (0.4%), an unexpected NH…mesityl clash emerges as dominant (26.1%). iSELF atomic decomposition identifies nitrogen (9.9 kcal mol^−1^) and catalyst fluorine (12.2 kcal mol^−1^) as primary steric contributors in the (*S*)-approach, a crucial N⋯F interaction completely overlooked in the original analysis.

While SELF elucidates steric mechanisms, complete understanding requires considering (among others) electrostatic and dispersion forces, whose interplay determines the overall energetic profile.

### 2.5 Unveiling the hidden dance of atropisomerism

Atropisomerism, the restricted rotation that creates axial chirality, is a cornerstone of asymmetric catalysis and drug design.^51,52^ With over 30% of FDA-approved drugs now containing atropisomeric scaffolds^52^ and the emergence of axially chiral ligands as privileged structures in catalysis,^51^ developing robust computational tools to predict and quantify rotational barriers has become crucial for rational molecular design.

However, understanding the true origin of rotational barriers can prove challenging: the molecule does not simply rotate, it ‘breathes'. In fact, when fragments rotate around an hindered σ-bond, they undergo spatial crowding while the connecting bond elongates (thereby relieving steric strain), which complicates the interpretation of energy landscapes. EDA and SAPT methods excel at dissecting intermolecular interactions, but they are inherently not suited for quantifying steric repulsion between covalently-connected fragments within a single molecule. This emphasizes the need for targeted tools to isolate and quantify pure steric effects.

**Fig. 7:**
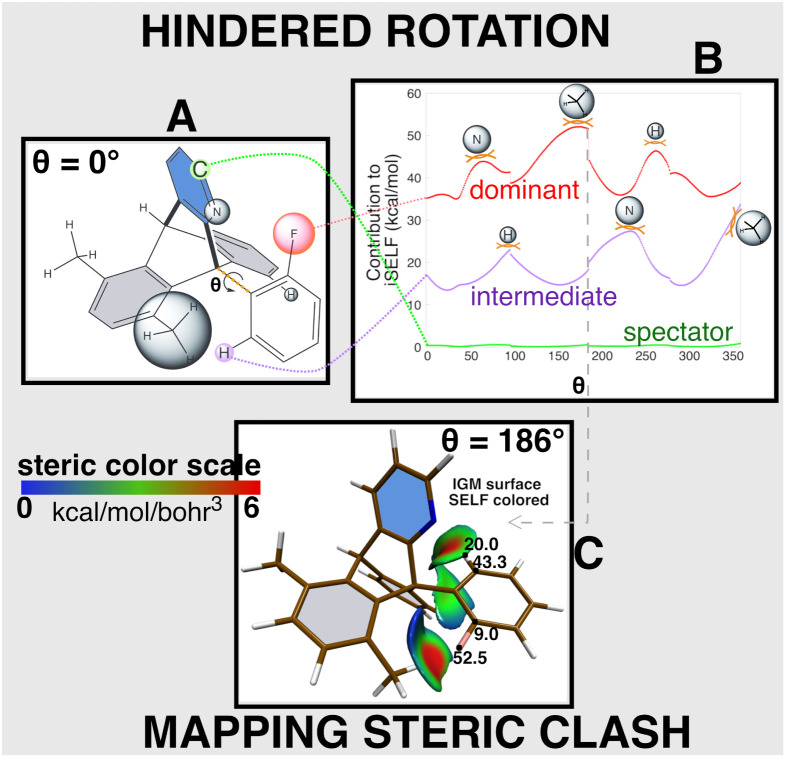
Atropisomerism in 9-arylazatriptycene;^53^ (A) molecular structure for *θ* = 0° with certain hydrogens omitted for clarity; the two SELF fragments are defined on either side of the rotatable C–C_Aryl_ bond; for the first fragment, the three chemical groups enclosed by grey spheres experience significant repulsive interactions during the rotation around the C-C_Aryl_ bond; (B) atomic iSELF contributions reported as a function of the rotation angle *θ* for three representative atoms (fluorin in red, hydrogen in purple, carbon in green color); (C) spatial characterization of steric clash between the two fragments on either side of the rotating C–C_Aryl_ bond for *θ* = 186°. 0.8 a.u.^−1^*δg*^inter^/*ρ* isosurface SELF colored in the range [0 : 0.6 kcal mol^−1^ bohr^−3^]. Atomic contributions in kcal mol^−1^. DFT calculations performed at the PBEPBE/6-31G* level of theory.

Using 9-arylazatriptycene^53^ as a model system (Fig. 7), we demonstrate how iSELF tracks the fluctuations of atomic contributions to steric repulsion during hindered rotation. The two sterically interacting fragments were defined as the molecular moieties on either side of the rotating C–C_Ar_ bond (without these two carbons). We performed a relaxed scan allowing molecular reorganization while constraining the dihedral angle *θ*. The iSELF approach distinguishes between spectator, intermediate, and key player atoms throughout the 360° rotation.

Three atoms exemplify this hierarchy: atom C (green) remains a spectator with minimal contribution, atom H (purple) shows moderate involvement, while fluorine (red) emerges as the dominant player, experiencing three distinct repulsion maxima when passing nitrogen (40–60°), methyl (170–190°), and hydrogen (260–280°) substituents.

At *θ* = 186°, although fluorine emerges as the dominant atomic contributor, the two bonds C–F (61.5 kcal mol^−1^) and C–H (63.3 kcal mol^−1^), contribute nearly equally (48.0% and 49.6% respectively) to the total interfragment repulsion (127.7 kcal mol^−1^ for each fragment) demonstrating the collective contribution of bonded atoms to steric hindrance.

An in-depth analysis of this system is provided in the SI (in Section S10).

For a more comprehensive appreciation of the steric repulsion intricacies, we invite readers to explore the animated evolution of SELF iso-surface during rotation (the video presented in the SI). This visualization provides a compelling and intuitive view of the subtle steric dynamics that occur over the course of the rotation.

### 2.6 From the Tolman angle to SELF: probing steric effects in Ni–phosphine complexes

To further demonstrate the SELF approach versatility, we performed a series of seven additional calculations on phosphine⋯nickel complexes of the type Ni(CO)_3_L (L = PR_1_R_2_R_3_, Ni(0)), directly inspired by Tolman's work.^5^ This new set of calculations tackles lone-pair availability in phosphines, a long-standing academic concern driven by steric considerations. In these complexes, we first computed the SELF-based steric interaction between the phosphine substituents (R_1_R_2_R_3_, fragment 1) and the three carbonyl ligands (fragment 2).

Our results reveal a compelling correlation between the Tolman cone angle and the integrated iSELF score (see Table 2 and Fig. S11 in SI).

**Table 2:** iSELF scores (kcal mol^−1^) and Tolman cone angles obtained for a series of seven nickel complexes Ni(CO)_3_L ((L = PR_1_R_2_R_3_) at equilibrium geometry. Level of theory: DFT BLYP/def2-TZVP, singlet state. Fragment 1 = substituents attached to phosphorus atom (R_1_R_2_R_3_)

Phosphine subst.	Tolman angle^a^ (°)	iSELF^b^	iSELF^c^
(NHCH_2_CH_2_)_3_	108	19.5	12.5
(Me)_3_	118	22.0	12.5
(O-*i*-Pr)_3_	130	65.9	28.6
Ph_2_(*i*-Pr)	150	73.3	28.5
(O-*t*-Bu)_3_	172	120.0	30.9
(*o*-Tol)_3_	194	125.2	44.6
(mesityl)_3_	212	170.2	50.2

a From ref. 5.

b Fragment 2 = (CO)_3_.

c Fragment 2 = Ni.

**Fig. 8:**
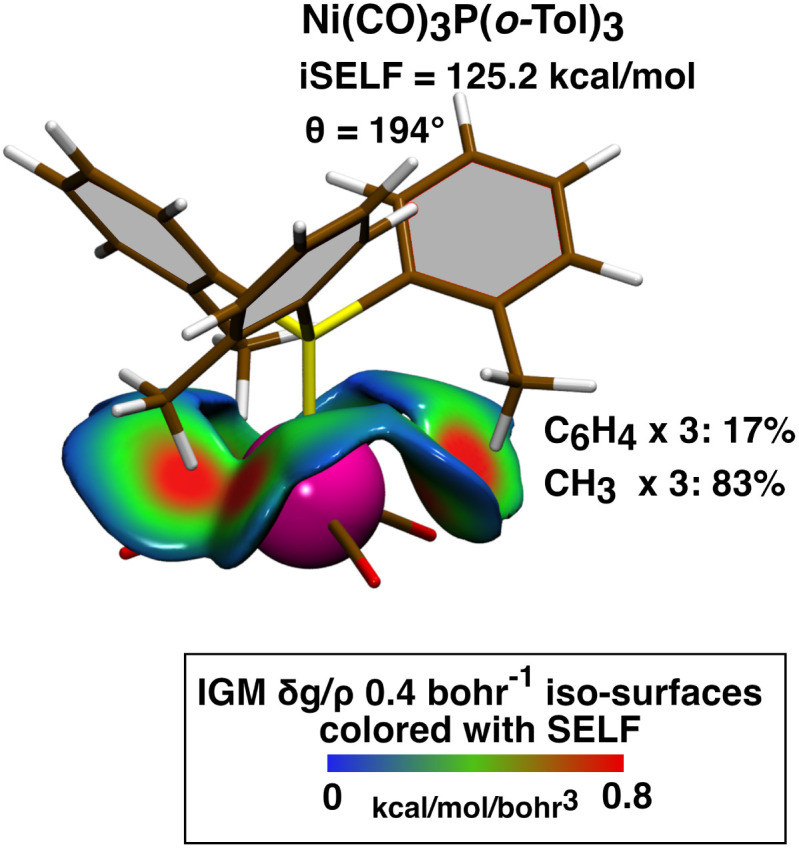
Steric analysis of Ni(CO)_3_P(*o*-Tol)_3_ at equilibrium geometry; Fragment 1 = (CO)_3_; Fragment 2 = ligands attached to phosphorus atom; 0.4 a.u.^−1^*δg*^inter^/*ρ* iso-surfaces colored by the SELF(**r**) values in the range 0 to 0.8 kcal mol^−1^ bohr^−3^ on a BGR color scale; atomic group decomposition in %. Level of theory: DFT BLYP/def2-TZVP, singlet state.

This agreement validates our quantum-mechanical approach while highlighting its key advantage over purely geometry-based models. Indeed, rooted in local electronic structure calculations, SELF provides a natural decomposition of steric demand, from individual atomic contributions to functional groups and entire molecular fragments (Fig. 8), which remains beyond the scope of Tolman's representation.

We also evaluated the steric repulsion between the phosphine substituents and the nickel center itself (score iSELF^*b*^ reported in Table 2). While the same trend with Tolman cone angles is observed, the magnitude of the interaction is notably smaller, revealing a metal more deeply ‘buried’ and thus less spatially exposed to the phosphine substituents than the carbonyl ligands.

### 2.7 Limitations, best practices

Diffuse basis functions may yield spurious SELF values, a well-recognized issue for some electronic descriptors in quantum chemistry. This limitation stems from the method's fundamental design: it performs fragment partitioning in Hilbert space rather than in real space. Apart from this diffuse basis set issue, the SELF method demonstrates robust consistency across different computational approaches and basis sets (see Tables S10 and S11 in SI), with coefficient of variation ranging from 5.1% for systems exhibiting representative intermediate steric repulsion to 9.1% for extreme steric clashes. Therefore, we recommend maintaining consistent computational parameters and emphasizing relative comparisons over absolute values, which is a standard quantum chemistry practice, while strictly avoiding diffuse orbitals in the atomic orbital basis set.

SELF requires atom-centered orbitals precluding its use with plane wave methods where atomic attribution is impossible.

Regarding the sensitivity of SELF to the choice of exchange-correlation functional in DFT calculations, we performed extra-calculations using a broad spectrum of DFT methods (see Table S12 in SI). We also systematically varied the dispersion correction scheme, including pairwise post-SCF methods (D3(BJ), D4) and self-consistent non-local approaches. Remarkably, the iSELF scores remain nearly constant across all tested combinations for the examined nitromethane⋯methanethiol complex (geometry taken from the NCIAtlas database,^54^ coefficient of variation of 1.2%). Similarly, B3LYP and M06-2X calculations on noble gas dimers yield virtually indistinguishable iSELF profiles (see Fig. S10 in SI), confirming that steric repulsion scores iSELF are largely decoupled from the dispersion treatment. In our view, this robustness can be rationalized on physical grounds: Pauli repulsion and London dispersion operate in fundamentally different spatial regimes, their distance dependence is very different. Steric repulsion manifests predominantly in regions of significant ED overlap, at short range. In contrast, dispersion interactions stems from longer-range correlation effects. Consequently, our recommendation is that the choice of dispersion correction scheme should be guided by energetic accuracy requirements rather than by considerations related to the SELF analysis, as the latter remains barely affected.

Regarding the question of the choice of exchange-correlation functional, despite the different levels of sophistication investigated (from LDA to double-hybrid functionals) the iSELF values remain remarkably similar. This striking consistency indicates that features captured by SELF are well converged across functional families, granting users considerable flexibility in their choice of method.

## 3 Conclusion

The Steric Exclusion Localization Function (SELF) provides a formally well-founded approach to one of the most fundamental interactions in chemistry: steric effects. Beyond three-dimensional mapping of repulsion regions, SELF enables rigorous quantification and atomic-level decomposition of steric interactions, capabilities that lie beyond the reach of traditional quantum-mechanical methods. Indeed, while EDA, SAPT, NBO and IQA methods quantify steric repulsion, to the best of our knowledge, they generally yield global energies without revealing where repulsion localizes or which atoms are responsible. Crucially, SELF extends its capabilities seamlessly from intermolecular contacts to intramolecular steric strain, an area where existing traditional methods struggle most. By unifying localization, quantification, and atomic attribution across these intra- and intermolecular contexts, SELF offers chemists an intuitive yet rigorous framework for dissecting steric interactions. From a conceptual standpoint, the present work suggests that steric effects may be rigorously defined as manifestations of Pauli kinetic energy excess arising from non-bonded repulsions, a physically grounded definition that could complement the current IUPAC framework, whose definition primarily describes the phenomenological manifestations of steric effects (on structure, reactivity) rather than their underlying physical origin. Fundamentally, SELF acts as an inter-fragment probe of the Fermi hole curvature. Importantly, SELF scores are not directly comparable in magnitude to SAPT or EDA exchange-repulsion energies. Indeed, SELF isolates the kinetic energy rise from Pauli exclusion using fully relaxed electron density, whereas SAPT/EDA report net exchange-repulsion effects from frozen fragment wavefunctions. Yet, both approaches yield consistent qualitative trends.

SELF represents a significant step toward making spatial steric analysis accessible to a broader chemical community, beyond specialized quantum chemists, offering both visualization capabilities and quantitative precision, which can guide chemical understanding, design, and education.

## Author contributions

Guillaume Hénon Just: formal analysis, methodology. Corentin Lefebvre: investigation, validation. Akil Rajamani: investigation, validation. Hassan Khartabil: investigation, validation, writing – review. Julien Pilmé: writing – review & editing, validation, conceptualization. E. Hénon: conceptualization, methodology, software, formal analysis, writing – original draft, visualization, project administration. All authors have given approval to the final version of the manuscript.

## Conflicts of interest

There are no conflicts to declare.

## Supplementary Material

SC-OLF-D5SC07952G-s001

SC-OLF-D5SC07952G-s002

SC-OLF-D5SC07952G-s003

## Data Availability

The code for SELF can be found at http://igmplot.univ-reims.fr with DOI https://doi.org/10.1002/jcc.27123. The version of the IGMPLot code employed for this study is version 3.17. The original data supporting this article are available in the main text and supporting information (SI). Supplementary information: the theoretical foundations and implementation of SELF, including its formal derivation, atomic decomposition, computational setup, validation against Pauli-based descriptors, and illustrative applications to steric effects in molecular and catalytic systems. A supplementary movie visually illustrates the SELF methodology, showing how steric repulsion is identified, localized and quantified in three dimensions. See DOI: https://doi.org/10.1039/d5sc07952g.
