# Supplementary material for: A visualizable and widely applicable steric repulsion descriptor for guiding experimental chemistry
Source: Chem Sci. 2026 Jan 9;17(11):5394–403. doi: 10.1039/d5sc07952g (PMC12853102; doi:10.1039/d5sc07952g)
Supplement: SC-017-D5SC07952G-s001 [file SC-017-D5SC07952G-s001.pdf]

Supplementary Information (SI) for Chemical Science.
This journal is © The Royal Society of Chemistry 2026

Electronic Supporting Information

A Visualizable and Widely Applicable Steric Repulsion Descriptor for Guiding Experimental Chemistry

Guillaume Hénon Just,[a] Corentin Lefebvre,[b] Akil Rajamani,[c] Hassan Khartabil,[c] Julien Pilmé,[d] Éric Hénon*[c]

December 21, 2025

[a] G. Hénon Just,
Ecole Nationale des Ponts et Chaussées, Institut Polytechnique de Paris, département IMI, 6 et 8 avenue Blaise-Pascal, Cité Descartes, Champs-sur-Marne, 77455 Marne-la-Vallée, France

[b] Dr C. Lefebvre
Laboratoire de Glycochimie et des Agroressources d'Amiens, UR 7378, Université de Picardie Jules Verne, 10 rue Baudelocque, Amiens, France

[c] Dr A. Rajamani, Dr H. Khartabil, Prof. E. Hénon*
Institut de Chimie Moléculaire de Reims (ICMR) UMR CNRS 7372, Université de Reims Champagne-Ardenne, Moulin de la Housse BP 1039, 51687 Reims CEDEX 2, France
E-mail: eric.henon@univ-reims.fr

[d] Dr. J. Pilmé
Laboratoire de Chimie Théorique, UMR 7616, Sorbonne Université, Paris, France

Contents

S1 Programs used **3**

S2 Pauli difference map **3**

S3 Comparison of dimer's $C(\mathbf{r})_{40}$ and $C(\mathbf{r})_{04}$ iso-surfaces with pure monomer's KEE iso-surfaces **3**

S4 Comparison of $C(\mathbf{r})_{22}$ with KEE difference map **4**

S5 Demonstration of the $c(\boldsymbol{r})$ formula and SELF atomic decomposition **5**
- S5.1 Complete atomic orbital expansion of the $c(\boldsymbol{r})$ formula . . . 5
- S5.2 Computational optimization via exact fragment formulation . . . 6
- S5.3 Atomic decomposition . . . 7
  - S5.3.1 Distribution Algorithm . . . 7
  - S5.3.2 Fragment 1 Analysis . . . 7
  - S5.3.3 Fragment 2 Analysis . . . 7
- S5.4 Weight Calculation . . . 8
- S5.5 Matrix Structure and Normalization . . . 8
- S5.6 Final Atomic Contributions . . . 8
- S5.7 Graphical Overview and Summary . . . 8

S6 Graphical representation of SELF **9**

S7 Contributions to the Pauli kinetic energy excess **9**
- S7.1 Contribution and energy analysis . . . 10
- S7.2 Geometries . . . 12

S8 Minimal input file setup for SELF calculations with the IGMPlot program **13**

S9 Rotational anisotropy of interfragment repulsion **13**

S10 Atropisomerism investigation through SELF analysis **15**

S11 Influence of the level of theory (theory and basis set) on iSELF **17**
- S11.1 Dimer 1, intermediate steric repulsion . . . 17
- S11.2 Dimer 2, extreme steric repulsion . . . 17

S12 Influence of the DFT functional and dispersion on iSELF **18**
- S12.1 Noble gas dimers . . . 18
- S12.2 nitromethane$\cdots$methanethiol complex . . . 19

S13 Steric effects in $Ni(CO)_3L$ complexes: correlation with Tolman cone angles **21**

S1 Programs used

- Gaussian[1] was employed to perform single point calculations on specific geometries to generate wave functions required for SELF calculations.
- IGMPlot[2] (version 3.17) was employed to perform SELF and IGM calculations.
- NCI-EDA program[3] was used to generate EDA-NCI data.
- PSI4[4] code was used to generate SAPT data.
- VMD software[5] was employed to visualize SELF results and generate figures.

S2 Pauli difference map

Pauli difference map was numerically obtained by subtracting the monomer's Kinetic Energy Excess (KEE) from the dimer's KEE. Electronic structures were obtained at the DFT M06-2X/def2-TZVP level of theory using the monomer's geometries from the water dimer structure, and exactly the same grid parameters for strict comparison (subtraction of cubes). The calculations were performed using the IGMPlot code.

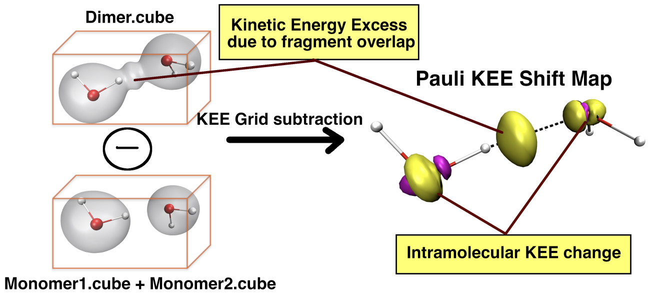

Figure S1 'Pauli Kinetic Difference Map' obtained for the water dimer by subtracting the Monomer's KEE cube (superposed) from the Dimer's KEE cube. KEE = Kinetic Energy Excess = $C(\boldsymbol{r})$; iso-value = 0.016 a.u.

S3 Comparison of dimer's $C(\mathbf{r})_{40}$ and $C(\mathbf{r})_{04}$ iso-surfaces with pure monomer's KEE iso-surfaces

The iso-surfaces of $C(\mathbf{r})$ obtained separately for each water monomer and iso-surfaces obtained from the dimer's functions $C(\mathbf{r})_{40}$ and $C(\mathbf{r})_{04}$ in the SELF formalism are barely distinguishable when superimposed. For clarity, we have zoomed into the envelopes to better visualize the near-perfect overlap of the two isosurfaces (pink for pure monomer's KEE, for functions $C(\mathbf{r})_{40}$ and $C(\mathbf{r})_{04}$) in the dimer. Electronic structures were obtained at the DFT M06-2X/def2-TZVP level of theory.

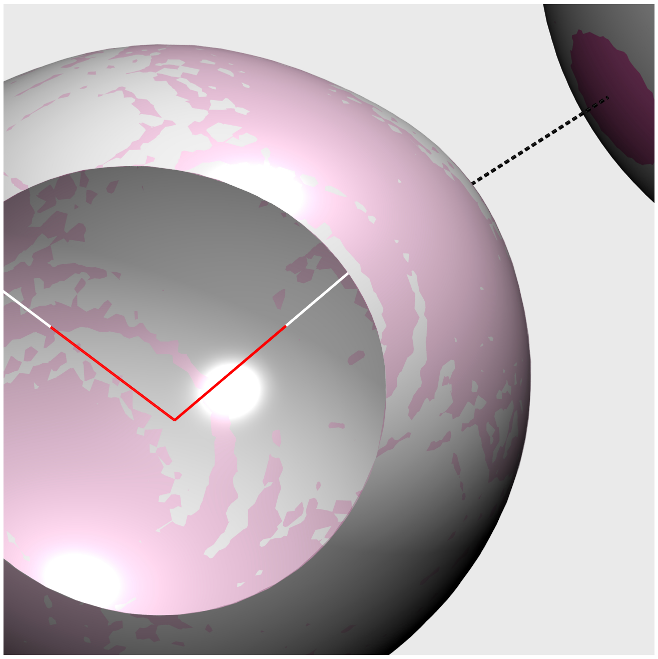

Figure S2 Comparison of pure monomer's Kinetic Energy Excess (KEE) (pink) with $C(\mathbf{r})_{40}$ and $C(\mathbf{r})_{04}$ iso-surfaces (gray) obtained from the SELF formalism (almost superimposed here); iso-value = 0.01 a.u.

S4 Comparison of $C(\mathbf{r})_{22}$ with KEE difference map

The iso-surface of the dimer's function $C(\mathbf{r})_{22}$ (gray) obtained in the SELF formalism and the iso-surface of Kinetic Energy Excess (KEE) difference of $C(\mathbf{r})$ (yellow) between monomer's and dimer cubes are barely distinguishable in between the two water molecules when superimposed. Electronic structures were obtained at the DFT M06-2X/def2-TZVP level of theory.

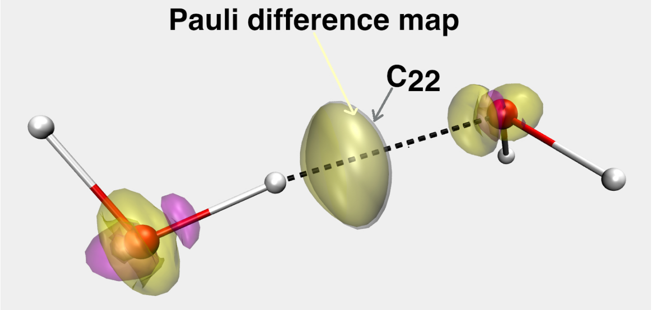

Figure S3 Comparison of the dimer's function $C(\mathbf{r})_{22}$ (gray) obtained from the SELF formalism with the map (yellow for positive and purple for negative contributions) derived from the manually computed KEE difference between dimer and monomers; iso-value = 0.01 a.u. for both iso-surfaces.

S5 Demonstration of the $c(\boldsymbol{r})$ formula and SELF atomic decomposition

At the heart of the SELF methodology is the quantity $c(\mathbf{r}) = 8\rho(\mathbf{r})G(\mathbf{r}) - |\nabla\rho(\mathbf{r})|^2$, with $\rho(\mathbf{r})$ the electron density and $G(\mathbf{r})$ the positive kinetic energy density. The SELF descriptor is given by $\text{SELF}(\mathbf{r}) = c(\mathbf{r})_{22}/(8\rho(\mathbf{r})) = C(\mathbf{r})_{22}$, with $c(\mathbf{r})_{22}$ is the part of $c(\mathbf{r})$ where two atomic orbitals (out of the four) belong to the first fragment, and the remaining two atomic orbitals belong to the second one.

S5.1 Complete atomic orbital expansion of the $c(\boldsymbol{r})$ formula

When molecular orbitals ψ_i are expressed as linear combinations of atomic orbitals φ_c:

$$\psi_i = \sum_c C_{ic}\varphi_c \tag{1}$$

the electron density $\rho = \sum_i n_i \psi_i^2$ expresses as:

$$\rho = \sum_{cd} D_{cd}\ \varphi_c\varphi_d \tag{2}$$

where D_{cd} is a term of the density matrix: $D_{cd} = \sum_i n_i C_{ic} C_{id}$, with fractional values of the molecular orbital occupancy numbers n_i. Using now the index pair a and c to facilitate index alignment in the subsequent demonstration, it comes:

$$\nabla\rho = 2\sum_{ac} D_{ac}\nabla\varphi_a\ \varphi_c \tag{3}$$

and using the additional index pair b and d:

$$|\nabla\rho|^2 = 4\sum_{abcd} D_{ac}D_{bd}\nabla\varphi_a.\nabla\varphi_b\ \varphi_c\ \varphi_d \tag{4}$$

In other respects, the gradient-based kinetic energy density $G = \frac{1}{2}\sum_i n_i \nabla\psi_i^\star.\nabla\psi_i$ expresses as:

$$G = \frac{1}{2}\sum_{ab} D_{ab}\nabla\varphi_a.\nabla\varphi_b \tag{5}$$

Then, using Equations 2 and 5:

$$8\rho G = 4\sum_{abcd} D_{ab}D_{cd}\nabla\varphi_a.\nabla\varphi_b\ \varphi_c\ \varphi_d \tag{6}$$

Hence, $c(\boldsymbol{r}) = 8\rho G - |\nabla\rho|^2$ expresses as:

$$c(\boldsymbol{r}) = 4\sum_{abcd}\left[D_{ab}D_{cd} - D_{ac}D_{bd}\right]\nabla\varphi_a.\nabla\varphi_b\ \varphi_c.\varphi_d \tag{7}$$

By retaining only the terms in which two atomic orbitals belong to the first fragment and the other two to the second fragment, we obtain the SELF score: $\text{SELF}(\mathbf{r}) = c(\mathbf{r})_{22}/(8\rho(\mathbf{r})) = C(\mathbf{r})_{22}$. This expression can be used for atomic decomposition as explained in the following.

S5.2 Computational optimization via exact fragment formulation

To save computational time, the ingredients for computing $c(\boldsymbol{r}) = 8\rho G - |\nabla\rho|^2$ can instead be determined as follows, considering two fragments 1 and 2:

$$\rho = \rho_1 + \rho_2 + 2\rho_{12} \tag{8}$$

$$\text{Electron density associated with fragment i: } \rho_i = \sum_{(ab)_{\in i}} D_{ab}\ \varphi_a\varphi_b$$

$$\text{Electron density shared between fragments i and j: } \rho_{ij} = \sum_{a_{\in i}}\sum_{b_{\in j}} D_{ab}\ \varphi_a\varphi_b$$

$$2G = 2G_1 + 2G_2 + 4G_{12} \tag{9}$$

$$\text{Twice the kinetic energy density associated with fragment i: } 2G_i = \sum_{(ab)_{\in i}} D_{ab}\ \nabla\varphi_a.\nabla\varphi_b$$

$$\text{Twice the kinetic energy density shared between fragments i and j: } 2G_{ij} = \sum_{a_{\in i}}\sum_{b_{\in j}} D_{ab}\ \nabla\varphi_a.\nabla\varphi_b$$

From there:

$$8\rho G = 4\left[\underbrace{2\rho_1 G_1 + 2\rho_2 G_2}_{40+04} + \underbrace{2\rho_1 G_2 + 2\rho_2 G_1 + 8\rho_{12} G_{12}}_{22} + \underbrace{4\rho_1 G_{12} + 4\rho_{12} G_1}_{31} + \underbrace{4\rho_2 G_{12} + 4\rho_{12} G_2}_{13}\right] \tag{10}$$

Considering now the electron density gradient used in the expression of $c(\boldsymbol{r})$:

$$\nabla\rho = \nabla\rho_1 + \nabla\rho_2 + 2\nabla\rho_{1'2} + 2\nabla\rho_{12'} \tag{11}$$

with:

$$\text{Electron density gradient associated with fragment i : } \nabla\rho_i = 2\sum_{(ab)_{\in i}} D_{ab}\ \varphi_b\nabla\varphi_a$$

$$\text{Electron density gradient shared between fragments i and j: } \nabla\rho_{i'j} = \sum_{a_{\in i}}\sum_{b_{\in j}} D_{ab}\ \varphi_b\nabla\varphi_a$$

Given this notation, we have:

$$|\nabla\rho|^2 = \underbrace{|\nabla\rho_1|^2 + |\nabla\rho_2|^2}_{40+04} + \underbrace{4|\nabla\rho_{1'2}|^2 + 4|\nabla\rho_{12'}|^2 + 2\nabla\rho_1\nabla\rho_2 + 8\nabla\rho_{1'2}.\nabla\rho_{12'}}_{22} +$$
$$\underbrace{4\nabla\rho_1.\nabla\rho_{1'2} + 4\nabla\rho_1.\nabla\rho_{12'}}_{31} + \underbrace{4\nabla\rho_2.\nabla\rho_{1'2} + 4\nabla\rho_2.\nabla\rho_{12'}}_{13}$$

The resulting expression for component 22 of $c(\boldsymbol{r})$ is:

$$c(\boldsymbol{r})_{22} = 2\rho_1 G_2 + 2\rho_2 G_1 + 8\rho_{12} G_{12} - (4|\boldsymbol{\nabla}\rho_{1'2}|^2 + 4|\boldsymbol{\nabla}\rho_{12'}|^2 + 2\boldsymbol{\nabla}\rho_1 \boldsymbol{\nabla}\rho_2 + 8\boldsymbol{\nabla}\rho_{1'2}.\boldsymbol{\nabla}\rho_{12'}) \tag{12}$$

This expression reduces computational complexity by limiting each component to at most two nested loops rather than four loop levels in the previous expression (Eq. 7). However, though saving time during calculations, it cannot be used for atomic decomposition. Therefore, the IGMPlot program includes two modes for calculating steric repulsion: SELF keyword (faster, but without atomic decomposition) and SELFATOMIC keyword (slower, but with atomic-level resolution).

S5.3 Atomic decomposition

We consider two interacting molecular fragments (i and j), each containing a specified number of atoms. At each node of the computational grid encompassing both fragments, we calculate a quantity termed $c(\boldsymbol{r})_{22}/(8\rho(\boldsymbol{r}))$, which represents the Pauli kinetic energy excess resulting from interaction between two atomic orbitals (AOs) from fragment 1 and two AOs from fragment 2. To achieve an atomic decomposition of the integrated score c_{22}, we construct a two-dimensional matrix **S**, where both dimensions span all atoms of (fragment 1 + fragment 2).

S5.3.1 Distribution Algorithm

For each grid node with a local c22(**r**) contribution, we apply the following distribution scheme.

S5.3.2 Fragment 1 Analysis

We first examine whether the two AOs from fragment 1 are localized on:

- **The same atom A**: the diagonal element S_{AA} is incremented by the c22(**r**)$/(8\rho(\mathbf{r}))$ score:

$$\mathbf{S}_{AA} = \mathbf{S}_{AA} + c(\boldsymbol{r})_{22}/(8\rho(\mathbf{r})) \tag{13}$$

$$\tag{14}$$

- **two different atoms A and B of fragment 1**: the c22(**r**)$/(8\rho(\mathbf{r}))$ score is distributed as follows:

$$\mathbf{S}_{AB} = \mathbf{S}_{AB} + c(\boldsymbol{r})_{22} \times w_A/(8\rho(\mathbf{r})) \tag{15}$$

$$\mathbf{S}_{BA} = \mathbf{S}_{BA} + c(\boldsymbol{r})_{22} \times w_B/(8\rho(\mathbf{r})) \tag{16}$$

Where w_A and w_B are atomic weights ($0 \leq w \leq 1$) satisfying $w_A + w_B = 1$.

S5.3.3 Fragment 2 Analysis

The same procedure is applied to fragment 2.

S5.4 Weight Calculation

The atomic weights w are computed as follows:

$$w_A = \frac{\nabla \rho_A}{\nabla \rho_A + \nabla \rho_B} \tag{17}$$

where $\nabla \rho_x$ represents the atomic gradient of electron density for atom x. These atomic gradients can be obtained either from the IGM (Independent Gradient Model) method using the Gradient-Based Partition (GBP) scheme or from the Hirshfeld partitioning scheme, both being implemented in IGMPlot. These weights measure the relative contribution of each atom of fragment 1 to c22($\mathbf{r}$).

S5.5 Matrix Structure and Normalization

The resulting matrix **S** encompasses all atoms from both fragments, with non-zero contributions appearing in two blocks corresponding to each fragment. Inter-fragment matrix elements remain zero since atoms cannot simultaneously belong to both fragments. The significance of the matrix element $\mathbf{S}_{AB}$ is as follows: it represents the contribution of atom A to the AB pair of fragment 1 in the overall interaction with fragment 2.

After scanning all nodes on the grid, the sum of all the matrix elements is twice the total integrated C_{22} score (since each local $C_{22}(\boldsymbol{r}) = c_{22}(\boldsymbol{r})/(8\rho(\mathbf{r}))$ has been added to the two blocks of the matrix **S**). Since each $C_{22}(\boldsymbol{r})$ local score contributes equivalently to both fragments, we normalize by dividing all matrix elements by two to recover the total inter-fragment repulsion interaction energy.

S5.6 Final Atomic Contributions

The contribution of any atom A of fragment 1 (or 2) to the total interaction is obtained by summing the corresponding row elements:

$$\text{Contribution}_A = \sum_{k \in 1 \text{ or } 2} \mathbf{S}_{Ak} \tag{18}$$

where k runs over atoms of the considered fragment. This provides the atomic decomposition of the inter-fragment interaction.

S5.7 Graphical Overview and Summary

A term $\mathrm{C}(\mathbf{r})_{22}$ involving four atoms (i, j in fragment A, k, l in fragment B) is distributed in the two inner fragment blocks of the matrix **S**. In the fragment A block, the elements S_{ij} and S_{ji} receive $w_i\mathrm{C}(\mathbf{r})_{22}/2$ and $w_j\mathrm{C}(\mathbf{r})_{22}/2$, respectively (the same allocation scheme applies in fragment B block). The atomic weights w are derived from a Hirshfeld-type partitioning scheme. The steric contribution of a given atom i (from fragment A) is the sum of all $\mathbf{S}_{ij}$ elements, where j runs over all atoms of fragment A.

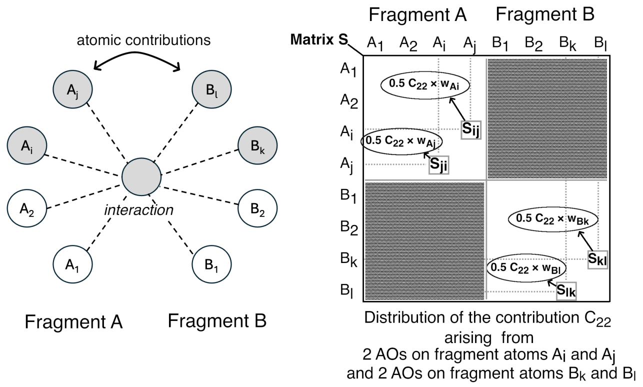

Figure S4 Schematic representation of the atomic decomposition scheme for the steric repulsion SELF.

S6 Graphical representation of SELF

To visualize steric effects spatially, we color-code the IGM $\delta g^{inter}/\rho$ iso-surface[6,7] with $SELF(\mathbf{r})$ values. This iso-surface captures ED overlap regions where steric repulsion may emerge, with BGR coloring revealing repulsion intensity (red = maximum).

In the IGM framework, while δg has units and varies greatly between strong covalent and weak non-covalent interactions (which is an advantage to characterize and quantify interactions), dividing by the electron density ρ: $\delta g/\rho$ produces a descriptor with a unified scale. This normalization enables catching at a glimpse the full spectrum from covalent to non-covalent regions without bias or threshold adjustments, though it precludes quantitative energy assessment. In this way, both strong and weak Pauli repulsions can be easily characterized and identified within a single calculation.

In this work, to avoid artifacts near vanishing density, we use $\delta g^{inter}/(\rho+\varepsilon)$ with $\varepsilon = 0.0005$ *a.u.*.

S7 Contributions to the Pauli kinetic energy excess

The water dimer has been examined at the (DFT) M06-2X/def2-TZVP level of theory for four geometries. First, the equilibrium geometry was investigated. Next, the structure was compressed from equilibrium geometry by bringing the two molecules closer together (see the three geometries below labelled '90%', '80%' and '70%').

A practical limitation of components involving fragment-interior regions (40, 04, 31, 13) is their relative marked grid sensitivity compared to 22 (see Table 1), stemming from their localization in high kinetic energy density regions near atomic nuclei.

S7.1 Contribution and energy analysis

Table S1 Equilibrium geometry of the water dimer taken from the S22 data set[8], with the original H-bond distance (1.952 Å). Decomposition of the kinetic energy excess due to Pauli repulsion between water molecules in the water dimer: influence of the IGMPlot grid stepsize (in Å) on the C_{xy} integrated contributions to C; kinetic energy reported in Hartee. (DFT) M06-2X/def2-TZVP level of theory. CPU time using 192 cores reported for indicative purpose.

Grid stepsize (Å)	40	04	31	13	22	C	time (s) for case 22
0.200	18.39174	19.15826	-0.00321	0.00246	0.12086	37.67011	< 1
0.100	18.58138	18.56066	-0.00340	0.00216	0.12084	37.26164	< 1
0.050	18.59094	18.56385	-0.00339	0.00214	0.12085	37.27439	6
0.025	18.59253	18.56385	-0.00339	0.00213	0.12085	37.27597	47
0.010	18.59253	18.56544	-0.00339	0.00213	0.12085	37.27756	717

Table S2 Geometry taken from the S22 data set[8], shifted in the H-bond axis to 90% of the original H-bond distance (1.756 Å). Decomposition of the kinetic energy excess due to Pauli repulsion between water molecules in the water dimer: influence of the IGMPlot grid stepsize (in Å) on the C_{xy} integrated contributions to C; kinetic energy reported in Hartee. (DFT) M06-2X/def2-TZVP level of theory.

Grid stepsize (Å)	40	04	31	13	22	C
0.200	18.39493	18.98137	-0.01391	-0.01424	0.17869	37.52684
0.100	18.59413	18.57978	-0.01361	-0.01451	0.17867	37.14579
0.050	18.61962	18.58616	-0.01359	-0.01449	0.17869	37.35639
0.025	18.62281	18.58775	-0.01359	-0.01450	0.17869	37.36116
0.010	18.62122	18.58775	-0.01359	-0.01450	0.17869	37.35957

Table S3 Geometry taken from the S22 data set[8], shifted in H-bond axis to 80% of the original H-bond distance (1.561 Å). Decomposition of the kinetic energy excess due to Pauli repulsion between water molecules in the water dimer: influence of the IGMPlot grid stepsize (in Å) on the C_{xy} integrated contributions to C; kinetic energy reported in Hartee. (DFT) M06-2X/def2-TZVP level of theory.

Grid stepsize (Å)	40	04	31	13	22	C
0.200	19.26184	18.35509	-0.04451	-0.03314	0.26253	37.80181
0.100	18.62600	18.67540	-0.04303	-0.03426	0.26234	37.48645
0.050	18.62759	18.67540	-0.04323	-0.03419	0.26234	37.48791
0.025	18.62919	18.67540	-0.04327	-0.03418	0.26234	37.48948
0.010	18.62919	18.67540	-0.04327	-0.03418	0.26234	37.48948

Table S4 Geometry taken from the S22 data set[8], shifted in H-bond axis to 70% of the original H-bond distance (1.366 Å). Decomposition of the kinetic energy excess due to Pauli repulsion between water molecules in the water dimer: influence of the IGMPlot grid stepsize (in Å) on the C_{xy} integrated contributions to C; kinetic energy reported in Hartee. (DFT) M06-2X/def2-TZVP level of theory.

Grid stepsize (Å)	40	04	31	13	22	C
0.200	19.34312	18.39174	-0.07700	-0.07199	0.38119	37.96706
0.100	18.68815	18.78217	-0.07423	-0.07436	0.38065	37.70238
0.050	18.68815	18.77102	-0.07449	-0.07415	0.38065	37.69118
0.025	18.68974	18.77261	-0.07456	-0.07412	0.38065	37.69432
0.010	18.68974	18.77261	-0.07455	-0.07412	0.38065	37.69433

Table S5 Energy analysis of the water dimer. Relative energies 'E' derived from (DFT) M06-2X/def2-TZVP computations. Boltzmann population calculated at 298 K. Integrated SELF contributions C_{xy} are reported as a function of geometric compression; for C_{31} and C_{13}, values in parentheses are % of C_{22}; 4 geometries considered: equilibrium structure taken from the S22 data set[8] and geometry shifted in H-bond axis to 90%, 80% and 70% of the original H-bond distance.

Compression level	Equil.	90%	80%	70%
H $\cdots$ O distance (Å)	1.952	1.756	1.561	1.366
E (kcal/mol)	0.0	0.6	3.6	11.8
Boltzmann pop. (%)	73.2	26.6	0.167	1.59E-7
C_{22}	75.8	112.1	164.6	238.9
$\|C_{31}\|$	1.3 (1.8%)	9.1 (8.1%)	21.4 (13.0%)	46.5 (19.5%)
$\|C_{13}\|$	2.1 (2.8%)	8.5 (7.6%)	27.2 (16.5%)	46.8 (19.6%)

We computed the partition function (Z = 1.363) from the exponential weighting of each four energy levels, then derived the fractional population of each state by normalizing its weight to the total.

Our analysis in four representative geometries: equilibrium, moderately compressed (+0.6 kcal/mol), pronounced compression (+3.6 kcal/mol) and highly distorted (+11.5 kcal/mol), demonstrates that the contributions of C31 and C13 remain consistently minor: <3%, <9%, <17% and <20% of C22, respectively. At room temperature, the populations of C31 and C13 are at least one order of magnitude lower than that of C22, as dictated by their limited thermal accessibility under the Boltzmann distribution.

S7.2 Geometries

Table S6 Equilibrium water dimer geometry employed (in Å)

Atom	X	Y	Z
O	-1.55101	-0.11452	0.00000
H	-1.93426	0.76250	0.00000
H	-0.59968	0.04071	0.00000
O	1.35062	0.11147	0.00000
H	1.68040	-0.37374	-0.75856
H	1.68040	-0.37374	0.75856

Table S7 Water dimer geometry shifted in H-bond axis to 90% of the original H-bond distance (in Å)

Atom	X	Y	Z
O	-0.95633	-0.12064	0.00000
H	-1.30753	0.76970	0.00000
H	0.00000	0.00000	0.00000
O	1.75643	0.00000	0.00000
H	2.06839	-0.49685	-0.75856
H	2.06839	-0.49685	0.75856

Table S8 Water dimer geometry shifted in H-bond axis to 80% of the original H-bond distance (in Å)

Atom	X	Y	Z
H	-0.59968	0.04071	0.00000
O	0.96056	0.09732	0.00000
H	1.29034	-0.38789	-0.75856
H	1.29034	-0.38789	0.75856
O	-1.55101	-0.11452	0.00000
H	-1.93426	0.76250	0.00000

Table S9 Water dimer geometry shifted in H-bond axis to 70% of the original H-bond distance (in Å)

Atom	X	Y	Z
H	-0.59968	0.04071	0.00000
O	0.76553	0.09024	0.00000
H	1.09531	-0.39497	0.75856
H	1.09531	-0.39497	-0.75856
O	-1.55101	-0.11452	0.00000
H	-1.93426	0.76250	0.00000

S8 Minimal input file setup for SELF calculations with the IGMPlot program

Minimal param.igm input file for a SELF calculation with atomic decomposition:

```
1
mol.wfx
FRAG1 1;2;4;5
FRAG2 7-10
SELFATOMIC
```

A .wfx file contains the complete wavefunction information from a quantum chemical calculation (GTO orbitals). Note that IGMPlot is also able to read .rkf files generated by ADF software (STO orbitals). FRAG1 and FRAG2 keywords are employed to specify the atoms of studied interacting fragments 1 and 2. Here, fragment 1 is made of atoms 1,2,4 and 5, while fragment 2 is made of atoms 7,8,9,10 of the system. Instead of SELFATOMIC keyword, the SELF keyword can be used to implement a simple and fast SELF analysis (without atomic decomposition). Beyond quantitative analysis (steric interaction energies and atomic contributions), IGMPlot program automatically provides a VMD script along with cube files to visualize steric clash regions in 3D space

S9 Rotational anisotropy of interfragment repulsion

In addition to the system presented in the main text, where a softer hydrogen atom was involved in steric repulsion, the $NH_3 \cdots$Cl-F dimer was selected to probe steric repulsion involving a hard atom (fluorine) approaching the nitrogen lone pair upon rotation. This configuration reveals instructive differences between SELF and the frozen-density EDA-NCI reference.

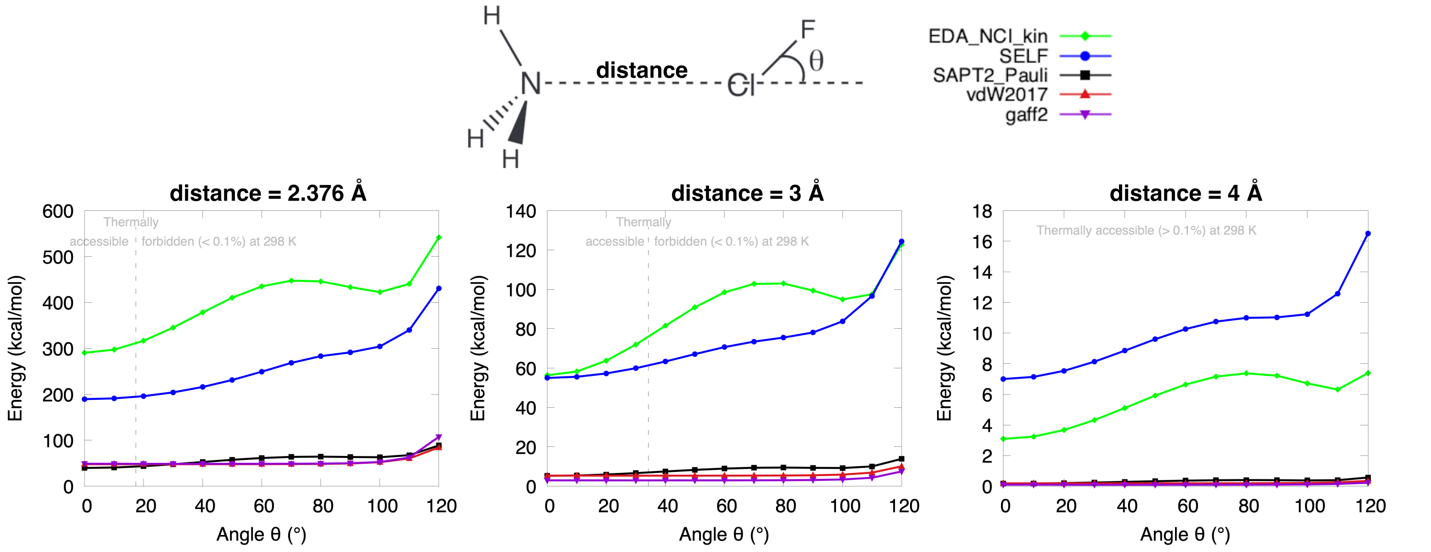

Figure S5 Repulsion energy analysis as a function of the tilt angle for the ammonia···ClF dimer, N···Cl distance fixed at either 2.376 Å, 3.000 Å, or 4.000 Å. EDA-NCI kinetic component (B3LYP/6-31G*, green), SELF (B3LYP/6-31G*, blue), SAPT2 (6-31G*, black), vdW2017 (red) and gaff2 (purple), with the two latter curves nearly coincident and visually hardly distinguishable.

At the largest N···Cl separation (4.000 Å, Fig. S5), the total interaction energies (encompassing both repulsive and attractive contributions, not reported on the plot) remain relatively modest, ranging from approximately -2.0 to +0.4 kcal/mol, corresponding to thermally accessible configurations. Under these conditions, the SELF and EDA-NCI curves exhibit relatively parallel profiles, with only modest separation throughout the angular scan. Interestingly, the SELF curve lies slightly above the EDA-NCI one. One tentative interpretation is that, when monomers are mutually attracted (low-repulsion regime), electronic relaxation may redistribute electron density (through polarization or charge transfer) in a way that does not systematically reduce (and may even slightly enhance) the overlap between electron clouds.

At the shortest N···Cl separation (2.376 Å, Fig. S5), the total interaction energy spans a much wider range, from -11 to $+31$ kcal/mol (not reported on the plot), giving rise to two distinct regimes: thermally accessible and thermally forbidden at 298 K. Steric repulsion component now reaches several hundred kcal/mol in both domains. Despite this significant increase in magnitude, the SELF and EDA-NCI curves retain similar shapes. However, the EDA-NCI repulsion consistently exceeds the SELF prediction by approximately 100 kcal/mol. In such strongly repulsive configurations, it is reasonable to expect that electronic relaxation naturally can reduce the overlap between electron clouds, thereby lowering steric repulsion. The frozen-density constraint in EDA-NCI prevents this relief, resulting in higher Pauli repulsion estimates compared to SELF.

Very interestingly, a subtle feature emerges for this interacting system across all three distances, though it is much more pronounced at $d = 3.000$ Å and $d = 2.376$ Å: the two curves are not strictly parallel in the central region of the plot. In the SELF profile near $\theta \approx 90$, a slight plateau appears (a deceleration in the steric repulsion increase), while EDA-NCI exhibits an even more pronounced feature. Indeed, very surprisingly, for the EDA-NCI curve, a steric repulsion decrease and even an actual local minimum are observed. This behavior is not an artifact but reflects the underlying electronic structure: as F rotates around Cl, the three lone pairs of Cl rotate concomitantly due to the covalent Cl–F bond. At $\theta = 90$, these lone pairs are oriented away from the N lone pair, momentarily compensating for the enhanced steric repulsion caused by the approaching F atom. This effect is detected by SELF but amplified in EDA-NCI analysis precisely because the frozen-density approximation cannot capture the smooth electronic relaxation that would attenuate such geometric

dependencies. This observation raises legitimate questions about the reliability of frozen-density methods as absolute references in such extreme interaction regimes. The presence of a σ-hole on chlorine adds another layer of complexity to this analysis, which lies beyond the scope of the present discussion.

S10 Atropisomerism investigation through SELF analysis

Using 9-arylazatriptycene as a model system[9] (**A** on Fig. S6), we demonstrate how SELF dissects the fundamental choreography of atropisomeric systems. The two sterically interacting fragments were defined as the molecular moieties on either side of the rotating C-C_{Ar} bond (without these two carbons). We performed a relaxed scan allowing molecular re-organization while constraining the dihedral angle θ.

Fig. S6 clearly demonstrates that the potential energy landscape (**B**, black curve) is dictated by the C-C_{Ar} rotatable bond distance (orange curve) as fragments rotate through 360°. This bond adapts dynamically throughout rotation, weakening in sterically congested situations to facilitate passage of flanking bulky groups, and strengthening otherwise (as highlighted by the C-C_{Ar} bond strength indicator IBSI[10] in green). Yet, the bond length fluctuations themselves are a direct manifestation of steric crowding. As the fluorinated aryl ring rotates, it encounters three main distinct energy barriers when the fluorine atom passes nitrogen ($\theta = 40°$), methyl (186°), and hydrogen (279°) substituents. To demonstrate the atomic resolution of the SELF approach, we focus here on tracking the fluorine atom's role in mediating steric repulsion between rotating fragments.

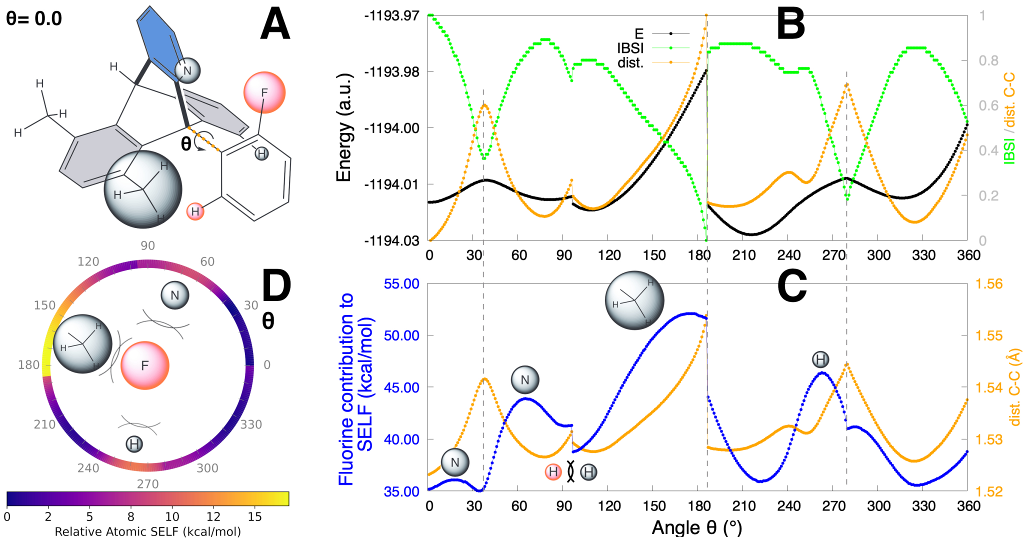

Figure S6 Atropisomerism in 9-arylazatriptycene[9]; Top left: molecular structure with some hydrogens omitted for clarity; the chemical groups enclosed by spheres indicate those involved in repulsive interactions during the rotation around the C-C_{Aryl} bond; three groups in grey belong to the first fragment, and two groups in pink to the second fragment; Top right: potential energy profile (black), C-C_{Aryl} bond length (orange) and bond strength (IBSI, green); Bottom right: atomic contribution of the fluorine atom to the steric repulsion measured by SELF (blue) and C-C_{Aryl} bond length (orange); Bond length and IBSI values are rescaled between 0 and 1 using min–max normalization; Bottom left: trigonometric circle, color-coded according to the repulsion energy barrier due to fluorine, as measured by the SELF analysis; DFT calculations at the PBEPBE/6-31G* level of theory. Note that although $\theta = 0°$ and 360° represent the same dihedral angle, relaxation at each step reveals distinct minima at these points (indicating that the reaction coordinate θ alone does not fully capture the complexity of the potential energy surface).

The key insight emerges from examining repulsive micro-events along the rotational pathway (**C** on Fig. S6):

fluorine-induced steric repulsion and molecular response exhibit a crucial phase shift — steric repulsion (blue curve) rises first, triggering subsequent C-C_{Ar} bond elongation (orange curve) and potential energy increase. This cause-and-effect sequence is particularly evident at the methyl (186°) and hydrogen (279°) barriers, where SELF peaks precede the potential energy response by several degrees.

The nitrogen-fluorine interaction showcases a striking phenomenon: an unexpected double-peak (18°, 60°) profile in the fluorine steric repulsion curve. As fluorine approaches nitrogen, the C-C_{Ar} bond stretches dramatically to relieve steric pressure, causing steric repulsion to paradoxically drop at the point of closest F···N approach (30°, calculations reveal that the F···N distance indeed increases at this point). This molecular overcompensation creates a repulsion minimum precisely where intuition predicts a maximum repulsion. Subsequently, the bond contracts and F···N steric repulsion resurges, reaching its true maximum (60°) only after fluorine has passed nitrogen, challenging the conventional understanding of steric interactions. The trigonometric steric map in Fig. S6 (**D**) summarizes these results, with fluorine experiencing three distinct repulsion maxima (+9, +17 and +11 kcal/mol).

Beyond individual atoms, SELF enables complete visualization of the steric landscape (Fig. S7). While conventional $\delta g^{\text{inter}}/\rho$ analysis shows all electronic overlap regions, SELF specifically highlights where Pauli repulsion dominates, a crucial distinction since not all close contacts are sterically significant. Readers can explore the animated evolution of SELF isosurface during rotation in the ESI for a compelling view of subtle repulsions throughout the rotation.

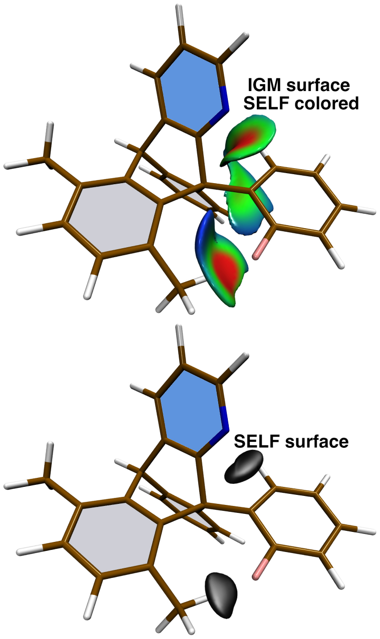

Figure S7 Atropisomerism in 9-arylazatriptycene, $\theta = 186°$; Top: 0.75 a.u. $\delta g^{inter}/\rho$ IGM isosurface colored by the $SELF(\mathbf{r})$ values in the range 0 to 6 kcal.mol^{-1}.bohr^{-3} on a BGR color scale (red = large steric interaction); Bottom: $SELF(\mathbf{r}) = 5$ kcal.mol^{-1}.bohr^{-3} isosurface; the two fragments used in this SELF analysis were defined on either side of the rotatable C-C_{Ar} bond; DFT calculations at the PBEPBE/6-31G* level of theory.

S11 Influence of the level of theory (theory and basis set) on iSELF

S11.1 Dimer 1, intermediate steric repulsion

The first example was taken from the NCIAtlas database.[12]

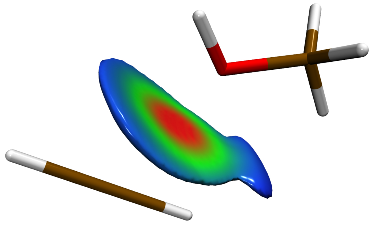

Figure S8 First example; 0.6 $\delta g^{inter}/\rho$ IGM isosurface colored by the $SELF(\mathbf{r})$ values in the range 0 to 3.6 kcal.mol^{-1}.bohr^{-3} on a BGR color scale (red = large steric interaction); Calculations performed at the HF/6-31G* level of theory.

Table S10 Molecular example exhibiting intermediate steric repulsion: study of the influence of the level of theory on the SELF values. Grid increments of 0.15 Å, using 192 CPU cores.

Level	iSELF (kcal/mol)	primitives	CPU time (s)
HF/6-31G*	65.9	136	< 1
HF/6-311G*	70.2	158	< 1
HF/def2-SVP	65.9	142	< 1
M06-2X/def2-SVP	61.7	142	< 1
M06-2X/def2-TZVP	70.0	252	2
wB97XD/def2-TZVP	68.1	252	2
M06-2X/cc-pVDZ	60.9	174	1
M06-2X/cc-pVTZ	65.0	310	3
MP2/def2-SVP	63.0	142	< 1
Mean	65.6		
Standard deviation	3.4		
Coefficient of Variation	5.1 %		

S11.2 Dimer 2, extreme steric repulsion

The second example was inspired by the atropisomerism investigation presented in the manuscript.

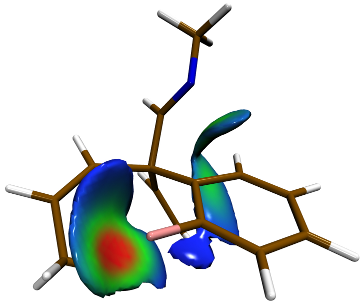

Figure S9 Second example; 0.6 $\delta g^{inter}/\rho$ IGM isosurface colored by the $SELF(\mathbf{r})$ values in the range 0 to 8 kcal.mol^{-1}.bohr^{-3} on a BGR color scale (red = large steric interaction); Calculations performed at the HF/6-31G* level of theory.

Table S11 Molecular example exhibiting extreme steric repulsion: study of the influence of the level of theory on the SELF values. Grid increments of 0.15 Å, using 192 CPU cores.

Level	SELF (kcal/mol)	primitives	CPU time (s)
HF/6-31G*	266.1	456	15
HF/6-311G*	294.6	528	25
HF/def2-SVP	286.1	462	16
M06-2X/def2-SVP	298.5	462	16
M06-2X/def2-TZVP	342.1	842	82
wB97XD/def2-TZVP	343.1	842	82
M06-2X/cc-pVDZ	268.2	574	30
M06-2X/cc-pVTZ	308.5	1000	114
MP2/def2-SVP	335.3	462	17
Mean	304.7		
Standard deviation	29.9		
Coefficient of Variation	9.8 %		

S12 Influence of the DFT functional and dispersion on iSELF

S12.1 Noble gas dimers

B3LYP and M06-2X calculations on noble gas dimers yield virtually indistinguishable iSELF profiles, suggesting that iSELF steric repulsion scores are largely decoupled from the dispersion treatment at short and long ranges.

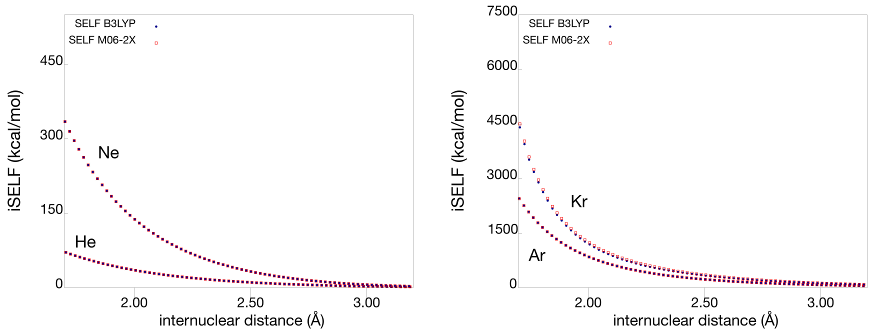

Figure S10 Steric repulsion in He, Ne, Ar and Kr homodimers; DFT B3LYP/6-31G* and M06-2X/6-31G* Levels of theory.

S12.2 nitromethane···methanethiol complex

Regarding the sensitivity of SELF to the choice of exchange-correlation functional, we performed extra-calculations using a broad spectrum of DFT methods (see Table S12 below). We also systematically varied the dispersion correction scheme, including pairwise post-SCF methods (D3(BJ), D4) and self-consistent non-local approaches. Remarkably, the iSELF scores remain nearly constant across all tested combinations for the examined nitromethane···methanethiol complex (coefficient of variation of 1.2%). As expected, post-SCF dispersion schemes such as D3(BJ) and D4, which add an energy correction without modifying the electron density, yield iSELF values strictly identical to their uncorrected counterparts. Self-consistent approaches incorporating non-local correlation into the SCF procedure (and thus directly shaping the electron density) induce only marginal variations. In our view, this robustness can be rationalized on physical grounds: Pauli repulsion and London dispersion operate in fundamentally different spatial regimes, their distance dependence is very different. Steric repulsion arises from the antisymmetry requirement of the wavefunction and manifests predominantly in regions of significant electron density overlap, where it induces an increase in kinetic energy (precisely what SELF captures). In contrast, dispersion interactions result from relatively long-ranged electron correlation effects. This spatial separation explains why dispersion corrections leave the SELF descriptor essentially unchanged: they simply do not affect the short-range region where Pauli repulsion dominates. Consequently, our recommendation is that the choice of dispersion correction scheme should be guided by energetic accuracy requirements rather than by considerations related to the SELF analysis, as the latter remains almost unaffected. Regarding the question of the choice of exchange-correlation functional, despite the different levels of sophistication investigated (from LDA to double-hybrid functionals) the iSELF values remain tightly close. This striking consistency indicates that features captured by SELF are well converged across functional families, granting users considerable flexibility.

Table S12 iSELF (kcal/mol) calculated for nitromethane ⋯ methanethiol complex with different DFT functionals and dispersion models. Fragment 1 = nitromethane, Fragment 2 = methanethiol. Basis set used: def2-TZVP.

Functional type	DFT level	Dispersion model	Determined	iSELF
LDA	PWLDA	none		94.2
	PWLDA-D3(BJ)	atom pairwise	post-scf	94.2
GGA	BLYP	none		92.0
	BLYP-D3(BJ)	atom pairwise	post-scf	92.0
meta-GGA	r^2SCAN	none		91.0
hybrid-GGA	B3LYP	none		92.6
	B3LYP-D3(BJ)	atom pairwise	post-scf	92.6
	B3LYP-D4	atom pairwise	post-scf	92.6
	B3LYP-SCNL	Non-Local variant of VV10	self-consistently	93.4
range-separated hybrid GGA	wB97X-D3(BJ)	atom pairwise	post-scf	92.3
	wB97X-V	Non-Local	self-consistently	92.3
range-separated hybrid meta-GGA	wB97M-V	Non-Local	self-consistently	93.7
double-hybrid GGA	DSD-PBEP86	none		94.7
			Mean	92.9
			Standard deviation	1.1
			Coeff. of variation	1.2%

All these calculations were performed using the ORCA package.[11]

The examined geometry was taken from the NCIAtlas database[12] (repulsion set):

Table S13 Compressed nitromethane ⋯ methanethiol geometry taken from the NCIAtlas database[12]

Atom	X	Y	Z
C	-1.498059411	-0.000000000	-1.600000000
H	-1.808519061	0.012758830	-2.641457161
H	-1.835724257	0.896679702	-1.094215496
H	-1.834939241	-0.909253068	-1.116129316
N	0.000000000	0.000000000	-1.600000000
O	0.555879823	-1.083121416	-1.626657474
O	0.555981287	1.083622540	-1.600000000
C	1.369281667	-0.000511580	2.752313725
H	1.349124288	-0.892054855	3.372130851
H	-1.007670843	0.000376478	2.433925637
H	1.349837282	0.892107772	3.372591573
H	2.286099248	-0.000543684	2.167565470
S	0.000000000	0.000000000	1.546700000

S13 Steric effects in $Ni(CO)_3L$ complexes: correlation with Tolman cone angles

To further demonstrate the versatility of the SELF approach, we performed a series of seven additional calculations on phosphine$\cdots$nickel complexes of the type $Ni(CO)_3L$ ($L = PR_1R_2R_3$, Ni(0)), directly inspired by Tolman's work.[13] This new set of calculations tackles lone-pair availability in phosphines, a long-standing academic concern driven by steric considerations. Through this work, we also show that our new SELF method applies to metal-containing systems.

In these complexes, we computed the SELF-based steric interaction between the phosphine substituents ($R_1R_2R_3$, fragment 1) and the three carbonyl ligands (fragment 2). Our results reveal a compelling correlation between the Tolman cone angle and the integrated iSELF score (see Table S14 and Fig. S11 below). However, considering the inherently simplified and empirical nature of the cone angle model, the Tolman approach cannot capture atomic or group details, compared to the SELF method.

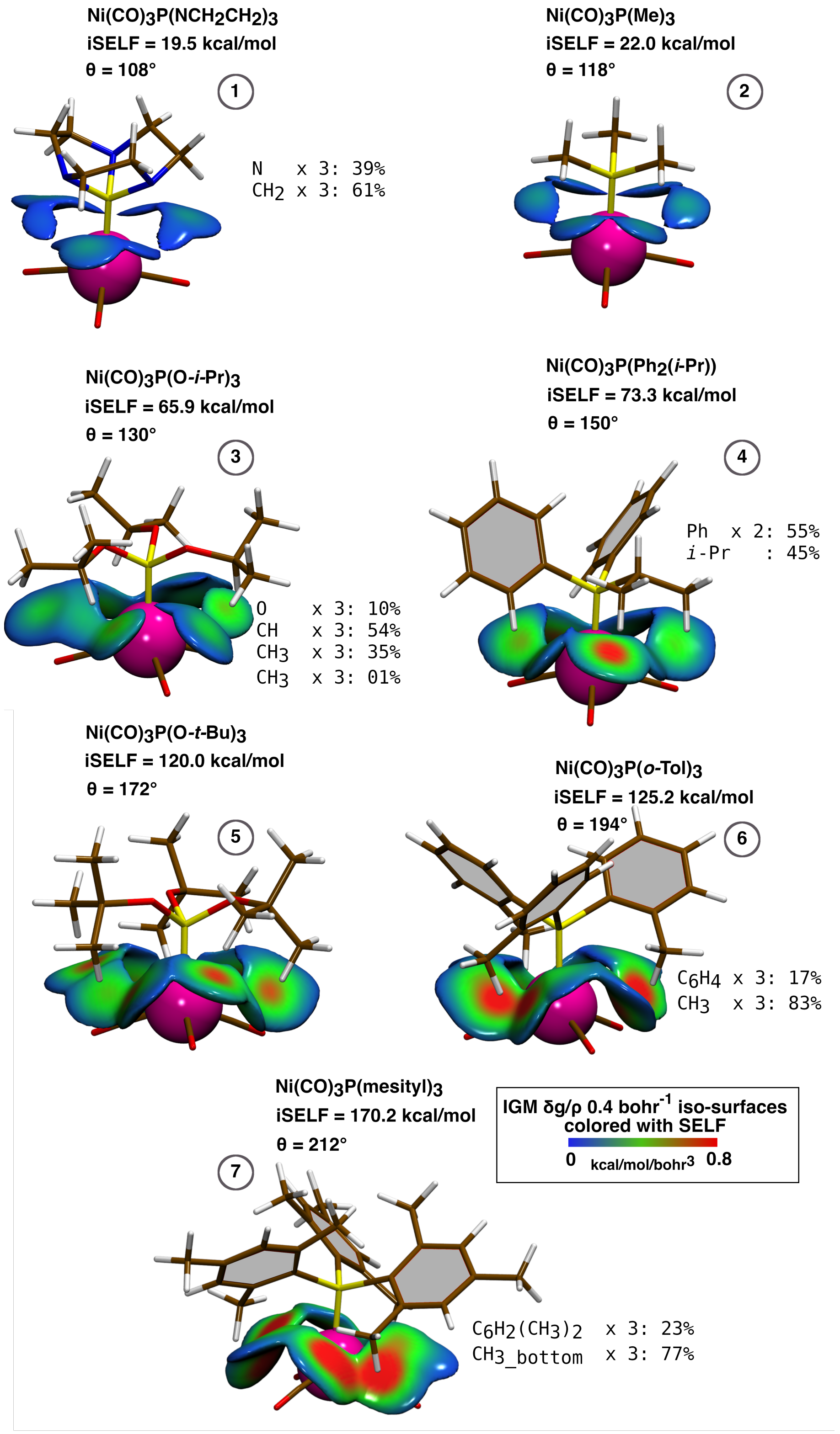

Figure S11 Steric analysis of a series of seven nickel complexes Ni(CO)$_3$L at equilibrium geometry; Fragment 1 = (CO)$_3$; Fragment 2 = ligands attached to phosphorus atom; 0.4 a.u.$^{-1}$ $\delta g^{inter}/\rho$ iso-surfaces colored by the SELF(r) values in the range 0 to 0.8 kcal.mol^{-1}.bohr^{-3} on a BGR color scale (the same $\delta g^{inter}/\rho$ iso-value and SELF range have been used throughout the series for homogenous visual comparison); atomic group decomposition in %. Level of theory: DFT BLYP/def2-TZVP, singlet state.

Table S14 iSELF scores and Tolman cone angles obtained for a series of seven nickel complexes Ni(CO)3L at equilibrium geometry. Level of theory: DFT BLYP/def2-TZVP, singlet state.

Molecule	Phosphine substituents	Tolman angle (°)	iSELF$^{(1)}$ (kcal/mol)	iSELF$^{(2)}$ (kcal/mol)
1	$(NHCH_2CH_2)_3$	108	19.5	12.5
2	$(Me)_3$	118	22.0	12.5
3	(O-*i*-Pr)$_3$	130	65.9	28.6
4	Ph_2(*i*-Pr)	150	73.3	28.5
5	(O-*t*-Bu)$_3$	172	120.0	30.9
6	(*o*-Tol)$_3$	194	125.2	44.6
7	(mesityl)$_3$	212	170.2	50.2

$^{(1)}$ Fragment 1 = $(CO)_3$; Fragment 2 = ligands attached to phosphorus atom.
$^{(2)}$ Fragment 1 = Ni; Fragment 2 = ligands attached to phosphorus atom.

Beyond predicting steric repulsion, SELF offers two key advantages over the Tolman approach: a full three-dimensional map of the steric interaction and an atom-by-atom decomposition of steric contributions (see Fig. S11). This provides direct insight into which specific atoms (or groups of atoms) dominate the steric pressure.

We also evaluated the steric repulsion between the phosphine substituents and the nickel center itself (score iSELF$^{(2)}$ reported in Table S14). While the same trend with Tolman cone angles is observed, the magnitude of the interaction is notably smaller, revealing a metal more deeply 'buried' and thus less spatially exposed to the substituents than the carbonyl ligands.

References

[1] M. J. Frisch, G. W. Trucks, H. B. Schlegel, G. E. Scuseria, M. A. Robb, J. R. Cheeseman, G. Scalmani, V. Barone, G. A. Petersson, H. Nakatsuji, X. Li, M. Caricato, A. V. Marenich, J. Bloino, B. G. Janesko, R. Gomperts, B. Mennucci, H. P. Hratchian, J. V. Ortiz, A. F. Izmaylov, J. L. Sonnenberg, D. Williams-Young, F. Ding, F. Lipparini, F. Egidi, J. Goings, B. Peng, A. Petrone, T. Henderson, D. Ranasinghe, V. G. Zakrzewski, J. Gao, N. Rega, G. Zheng, W. Liang, M. Hada, M. Ehara, K. Toyota, R. Fukuda, J. Hasegawa, M. Ishida, T. Nakajima, Y. Honda, O. Kitao, H. Nakai, T. Vreven, K. Throssell, J. A. Montgomery, Jr., J. E. Peralta, F. Ogliaro, M. J. Bearpark, J. J. Heyd, E. N. Brothers, K. N. Kudin, V. N. Staroverov, T. A. Keith, R. Kobayashi, J. Normand, K. Raghavachari, A. P. Rendell, J. C. Burant, S. S. Iyengar, J. Tomasi, M. Cossi, J. M. Millam, M. Klene, C. Adamo, R. Cammi, J. W. Ochterski, R. L. Martin, K. Morokuma, O. Farkas, J. B. Foresman and D. J. Fox, *Gaussian16 Revision C.01; Gaussian, Inc.: Wallingford, CT*, 2016, Gaussian Inc. Wallingford CT.

[2] C. Lefebvre, J. Klein, H. Khartabil, J.-C. Boisson and E. Hénon, *J. Comput. Chem.*, 2023, **44**, 1750–1766.

[3] M. Mandado and J. M. Hermida-Ramón, *J. Chem. Theory Comput.*, 2011, **7**, 633–641.

[4] D. G. Smith, L. A. Burns, A. C. Simmonett, R. M. Parrish, M. C. Schieber, R. Galvelis, P. Kraus, H. Kruse, R. Di Remigio, A. Alenaizan *et al.*, *J. Chem. Phys.*, 2020, **152**, year.

[5] W. Humphrey, A. Dalke and K. Schulten, *J. Mol. Graphics*, 1996, **14**, 33–38.

[6] C. Lefebvre, G. Rubez, H. Khartabil, J.-C. Boisson, J. Contreras-García and E. Hénon, *Phys. Chem. Chem. Phys.*, 2017, **19**, 17928–17936.

[7] C. Lefebvre, H. Khartabil, J.-C. Boisson, J. Contreras-García, J.-P. Piquemal and E. Hénon, *Chem. Phys. Chem.*, 2018, **19**, 724–735.

[8] P. Jurečka, J. Šponer, J. Černý and P. Hobza, *Phys. Chem. Chem. Phys.*, 2006, **8**, 1985–1993.
[9] A. B. Saida, D. Mahaut, N. Tumanov, J. Wouters, B. Champagne, N. Vanthuyne, R. Robiette and G. Berionni, *Angew. Chem. Int. Ed.*, 2024, **63**, e202407503.
[10] J. Klein, H. Khartabil, J.-C. Boisson, J. Contreras-García, J.-P. Piquemal and E. Hénon, *J. Phys. Chem. A*, 2020, **124**, 1850–1860.
[11] F. Neese, *Wiley Interdiscip. Rev. Comput. Mol. Sci.*, 2012, **2**, 73–78.
[12] K. Kriz, M. Novacek and J. Rezac, *Journal of Chemical Theory and Computation*, 2021, **17**, 1548–1561.
[13] C. A. Tolman, *Chem. Rev.*, 1977, **77**, 313–348.
