# Supplementary material for: A visualizable and widely applicable steric repulsion descriptor for guiding experimental chemistry
Source: Chem Sci. 2026 Jan 9;17(11):5394–403. doi: 10.1039/d5sc07952g (PMC12853102; doi:10.1039/d5sc07952g)
Supplement: SC-017-D5SC07952G-s002 [file SC-017-D5SC07952G-s002.pdf]

Supplementary Information (SI) for Chemical Science.
This journal is © The Royal Society of Chemistry 2026

Electronic Supplementary Information (ESI)

Click here to access the video:

http://igmplot.univ-reims.fr/videos/34_RobiettemovieSELF_3x.mp4

illustrating steric dynamics across hindered rotation:

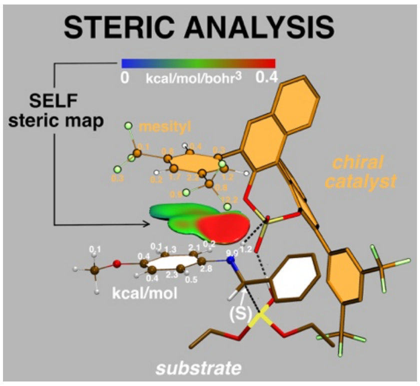

For a more comprehensive appreciation of the steric repulsion intricacies, we invite readers to explore the animated evolution of SELF iso-surface during rotation (presented in the ESI). This visualization provides a compelling and intuitive view of the subtle steric dynamics that occur over the course of the rotation.
